# Cholestenoic acid, an endogenous cholesterol metabolite, is a potent γ-secretase modulator

**DOI:** 10.1186/s13024-015-0021-z

**Published:** 2015-07-14

**Authors:** Joo In Jung, Ashleigh R. Price, Thomas B. Ladd, Yong Ran, Hyo-Jin Park, Carolina Ceballos-Diaz, Lisa A. Smithson, Günther Hochhaus, Yufei Tang, Rajender Akula, Saritha Ba, Edward H. Koo, Gideon Shapiro, Kevin M. Felsenstein, Todd E. Golde

**Affiliations:** Center for Translational Research in Neurodegenerative Disease, University of Florida, Gainesville, FL 32610 USA; Department of Neuroscience, University of Florida, Gainesville, FL 32610 USA; McKnight Brain Institute, College of Medicine, University of Florida, Gainesville, FL 32610 USA; College of Pharmacy, University of Florida, Gainesville, FL 32610 USA; SAI Life Sciences Ltd., Turkapally, AP500078 India; Department of Neuroscience, University of California, La Jolla, San Diego, CA 92093 USA; Pharmore, Inc., Gainesville, FL 32653 USA; Departments of Medicine and Physiology, Yong Loo Lin School of Medicine, National University of Singapore, Singapore, 119077 Singapore

**Keywords:** Cholestenoic acid, γ-secretase modulator (GSM), Amyloid, Alzheimer disease, Cholesterol, Steroid, Bile acid, Cytochrome P450

## Abstract

**Background:**

Amyloid-β (Aβ) 42 has been implicated as the initiating molecule in the pathogenesis of Alzheimer’s disease (AD); thus, therapeutic strategies that target Aβ42 are of great interest. γ-Secretase modulators (GSMs) are small molecules that selectively decrease Aβ42. We have previously reported that many acidic steroids are GSMs with potencies ranging in the low to mid micromolar concentration with 5β-cholanic acid being the most potent steroid identified GSM with half maximal effective concentration (EC_50_) of 5.7 μM.

**Results:**

We find that the endogenous cholesterol metabolite, 3β-hydroxy-5-cholestenoic acid (CA), is a steroid GSM with enhanced potency (EC_50_ of 250 nM) relative to 5β-cholanic acid. CA i) is found in human plasma at ~100-300 nM concentrations ii) has the typical acidic GSM signature of decreasing Aβ42 and increasing Aβ38 levels iii) is active in *in vitro* γ-secretase assay iv) is made in the brain. To test if CA acts as an endogenous GSM, we used Cyp27a1 knockout (Cyp27a1−/−) and Cyp7b1 knockout (Cyp7b1−/−) mice to investigate if manipulation of cholesterol metabolism pathways relevant to CA formation would affect brain Aβ42 levels. Our data show that Cyp27a1−/− had increased brain Aβ42, whereas Cyp7b1−/− mice had decreased brain Aβ42 levels; however, peripheral dosing of up to 100 mg/kg CA did not affect brain Aβ levels. Structure-activity relationship (SAR) studies with multiple known and novel CA analogs studies failed to reveal CA analogs with increased potency.

**Conclusion:**

These data suggest that CA may act as an endogenous GSM within the brain. Although it is conceptually attractive to try and increase the levels of CA in the brain for prevention of AD, our data suggest that this will not be easily accomplished.

**Electronic supplementary material:**

The online version of this article (doi:10.1186/s13024-015-0021-z) contains supplementary material, which is available to authorized users.

## Background

Accumulation of aggregated amyloid β peptides (Aβ) in the brain is proposed to be a key trigger in a complex neuropathological cascade that leads to Alzheimer’s disease (AD). Aβ is produced from the amyloid precursor protein (APP) through sequential proteolytic cleavages [1]. APP is first cleaved by β-secretase to produce a soluble APPβ and a membrane anchored APP carboxyl terminal fragment (CTFβ). The CTFβ is then cleaved by γ-secretase to produce extracellular Aβ peptides and APP-intracellular domain (AICD) fragments. Notably, a number of Aβ peptides are normally produced, with Aβ40 being the most abundant species with minor species including, but not limited to, Aβ37, 38, 39 and 42 routinely observed in most studies. These various species are not produced by simple classic endoproteolysis at multiple sites, but appear to arise from both variation in the initial substrate cleavage site which produces longer Aβs (i.e., Aβ48, Aβ49, and Aβ51) and the cognate AICD, which is then followed by multiple cycles of step-wise, carboxyl-peptidase like cleavages, all of which are mediated by γ-secretase. Although all Aβ peptides normally produced appear to accumulate in the human AD brain, the minor Aβ42 species is typically the most prevalent form that accumulates in the brain parenchyma [2, 3]. Additional lines of evidence further support the concept Aβ42 is the most pathogenic isoform [4], whereas Aβ40 may, under some circumstances, be a protective isoform [5, 6]. Many early onset familial AD (FAD) mutations linked with APP and Presenilin (PSEN, the catalytic subunit of γ-secretase) increase the relative levels of Aβ42 [7–10]. *In vitro* studies show that Aβ1-42 has a much stronger tendency to aggregate than Aβ1-40 [11]. In AD mouse model, Aβ42 plays a role as a seeding molecule for amyloid deposition but Aβ40 [6] does not. In fact, Aβ40 appears to prevent mice from amyloid deposition [5, 12]. Moreover, Aβx-42 is the earliest detectable Aβ isoform in the brain parenchyma [13–16]. The role of other shorter carboxyl-terminal truncated species is at this point unclear, though it is hypothesized that they may behave like Aβ40 [5, 17]. Altogether, there is ample rationale that decreasing the levels of Aβ42 could be a prophylactic approach to prevent accumulation of Aβ and, thereby, delay or prevent the development of AD.

There have been studies demonstrating that production and processing of Aβ can be influenced by membrane lipid composition [18–21]. In particular, membrane cholesterol appears to play an important role [18]. APP-CTFβ and γ-secretase are found in lipid rafts, composed primarily of cholesterol [18]. Further, it has been shown that cholesterol directly binds to the APP-CTFβ substrate [22, 23]. The interdependent interactions among the three components (APP-CTFβ, γ-secretase, and cholesterol) are postulated to create the optimal microenvironment for Aβ production. Indeed, it has been reported that γ-secretase activity is largely dependent on the amount of cholesterol, which affects Aβ production as a result [18, 24] though others have not reproduced this finding [25]. These observations suggest the potential for modulating γ-secretase activity and thus altering the overall Aβ levels or the ratios of Aβ isoforms produced by steroid derivatives as cholesterol surrogates.

Previously, we have reported steroid carboxylic acid γ-secretase modulators (GSMs) [26]. Numerous acidic steroids decrease Aβ42 levels and increase Aβ38 levels without changing total Aβ or Aβ40 levels [26]. Acidic steroid GSMs have gross structural similarity to the established-NSAID based GSMs in that a carboxylic acid group, that is key for GSM activity, is attached by a carbon tether chain to a highly lipophilic core structure [26, 27]. 5β-Cholanic acid (ursocholanic acid) was the most potent steroid GSM identified in our previous study with an EC_50_ of 5.7 μM, but the endogenous bile acids, lithocholic acid and ursocholic acid, were also found to be GSMs [26]. Mechanistically, GSMs decrease production of Aβ42 selectively by promoting step-wise γ-secretase cleavage and, thus, inherently increase shorter Aβ peptides [28, 29]. Because γ-secretase cleavage activity participates in a broad spectrum of cellular signaling mechanisms (i.e., Notch-1) [30], indiscriminate inhibition of γ-secretase activity has been essentially abandoned as a therapeutic approach for AD due to debilitating side effects associated with target-based toxicity. In contrast, GSMs do not alter overall γ-secretase activity, appear to be relatively selective for APP, and are, therefore, thought to be an intrinsically safe mechanistic approach to AD therapy; however, it has been challenging to identify GSMs that are potent, have sufficient brain penetrance, and lack off-target toxicity.

Considering that GSMs derived from synthetic compounds have toxicity issues that are not associated with target-based toxicity, we have explored whether other naturally occurring acidic steroids might have sufficient potency to be therapeutically useful. An extended screening identified 3β-hydroxy-5-cholestenoic acid (CA) as a highly potent GSM with an EC_50_ for Aβ42 lowering of 250 nM. As CA is produced endogenously during the course of cholesterol elimination in many extrahepatic organs including the brain [31, 32] and is present in human plasma at concentrations near its EC_50_ for GSM activity, we explored whether CA might function endogenously as a GSM. Our results showed that Cyp27a1−/− [33, 34] and Cyp7b1−/− [35] mice that reduce or increase brain CA, respectively, resulted in the predicted brain Aβ42 changes consistent with the hypothesis that CA is an endogenous GSM. Peripheral dosing of CA in wild type mice dramatically increased plasma CA levels, but not brain Aβ levels, suggesting limited brain exposure of peripheral CA. Structure-activity relationship (SAR) with multiple known and novel CA analogs studies failed to reveal CA analogs with increased potency. These studies show that though CA is a potent GSM that may act within the brain to regulate Aβ42 levels, exogenous administration of CA is not likely to be therapeutically useful for lowering Aβ42.

## Results

### CA is a potent GSM

**Fig. 1:**
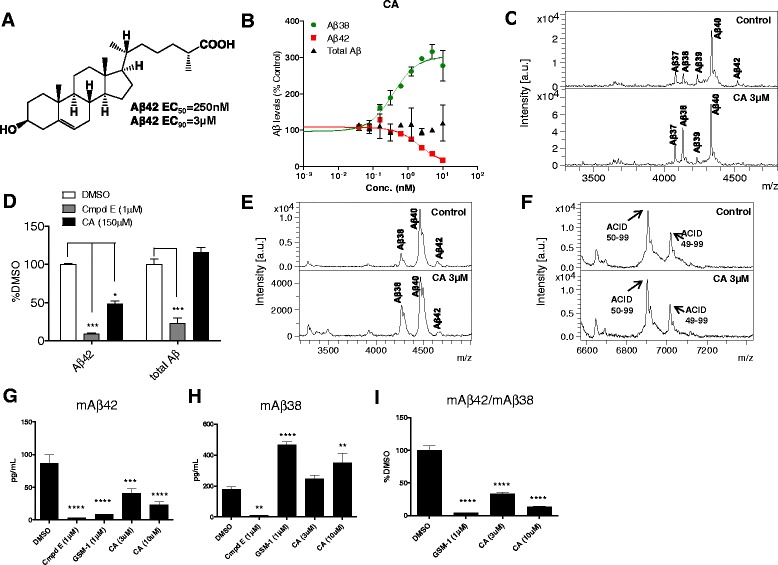
γ-Secretase modulatory effect of CA. **a** Chemical structure of CA with EC_50_ and EC_90_ for decreasing Aβ42 is illustrated. **b** Dose response curves of CA for Aβ42, Aβ38, and total Aβ in CHO-2B7 cells are shown. The Aβ levels in the conditioned media of the cells treated with CA at nine dose points for 16 h were measured by Aβ specific sandwich ELISAs. Aβ42 (red line) levels increase with the concentration, whereas Aβ38 (green line) levels decrease with the concentration. Total Aβ (black line) levels did not show significant changes. **c** Aβ spectra are illustrated by mass spectrometry after CA treatment at 3 μM in CHO-2B7 cells. Control refers to the conditioned media treated with DMSO in the cells, a solvent for CA. CA treatment at 3 μM increased Aβ38 peak and decreased Aβ42 peak with no significant changes in Aβ40 peak compared to the DMSO control. Identified Aβ peptides are indicated above the peaks. **d** *In vitro* γ-secretase assays show the direct effect of CA in γ-secretase modulation analyzed by ELISAs. Cmpd E is an irreversible *pan* γ-secretase inhibitor, which limits γ-secretase activity at the starting time point of the assay. The total γ-secretase activity was measured after 2 h of DMSO (solvent control) and CA incubation. Compared to the control, CA at 150 μM decreased Aβ42 by 50 % (*n* = 2 per group, repeated 2–3 times). **e** Aβ spectra obtained from MALDI-TOF mass spectrometry studies show that 20 μM CA from *in vitro* study decrease Aβ42 peak and increase Aβ38 peak compared to the DMSO treated control group. **f** For AICD spectra, AICD49-99 and AICD50-99 are presented as the dominant isoforms in both DMSO control and 20 μM CA treated groups. **g-i **The effects of CA as a GSM are shown in primary neuron-glia culture (*n* = 6). Mouse endogenous Aβ (mAβ) levels were measured by sandwich ELISAs. Cmpd E (γ-secretase inhibitor) decreased overall Aβ production. GSM-1, an acidic type GSM, was used as a positive control. For the primary neuron-glia culture, GSM-1 at 1 μM decreased level of mAβ42, but increased mAβ38 level. CA at 3 μM and 10 μM presented dose-dependent effects for decreasing mAβ42 and increasing mAβ38. Results were analyzed by two-way analysis of variance (ANOVA) followed by bonferroni post-hoc testing for group differences (Fig. 1d) and one-way analysis of variance (ANOVA) followed by Dunnett’s multiple comparisons (Fig. 1g-i). (***p < 0.001, **p < 0.01, *p < 0.5)

Based on previous studies that showed a number of acidic steroids are GSMs [26], we continued to test other additional acidic steroids for GSM activity. These studies revealed that the endogenous cholesterol metabolite CA (Fig. 1a) had potent GSM activity. In cell-based assays, the EC_50_ value for decreasing Aβ42 levels was 250 nM and, consistent with other acidic steroid GSMs, Aβ38 increased without alterations in total Aβ (Fig. 1b). CA’s GSM activity was further confirmed by IP/MS analysis using the conditioned media produced from CHO2B7 cells, which revealed selective lowering of Aβ42 and increased Aβ38 (Fig. 1c). To assess whether CA has a direct effect on γ-secretase, we performed *in vitro* γ-secretase cleavage assays (Fig. 1d-f). In these assays CA treatment decreased Aβ42 production by 51 % without any significant changes in total Aβ levels when the Aβ levels were compared to DMSO control (Fig. 1d). Compound E (Cmpd E), a non-selective γ-secretase inhibitor, decreased both Aβ42 (91 %) and total Aβ (77 %) production significantly (Fig. 1d). These Aβ levels demonstrate the initial Aβ levels in the assays. IP/MS Aβ and AICD profiles from the *in vitro* assay are illustrated in Fig. 1e and f, respectively. Again CA decreased Aβ42 and increased Aβ38 (Fig. 1e) and, as noted with previous studies of GSMs, did not affect ε-site utilizations (AICD49-99 and AICD50-99) (Fig. 1f). Next, we utilized primary mouse postnatal day 0 (P0) neuron-glia cultures of wild-type mice to determine the effect of CA on endogenous mouse Aβ (mAβ) levels (Fig. 1g-j). CA decreased mAβ42 production by ~60 % at 3 μM and by ~75 % at 10 μM (Fig. 1g) and increased Aβ38 production at 10 μM (Fig. 1h) resulting in a significant decrease in the Aβ42:Aβ38 ratio at both concentrations (Fig. 1l). Thus, confirming that CA acts as GSM on primary brain cells.

### Loss of Cyp27a1 and Cyp7b1 alters mouse brain Aβ42

**Fig. 2:**
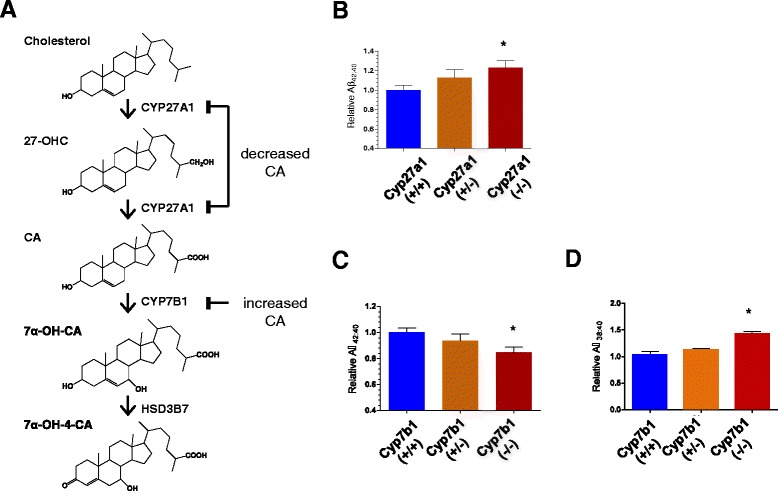
The effects of Cyp27a1 and Cyp7b1 genetic reductions on brain mAβ levels. **a** CA is found in the acidic cholesterol elimination pathway. Cholesterol is hydrolyzed by Cyp27a1 to produce 27-hydroxycholesterol (27-OHC) and CA sequentially. CA is further metabolized by Cyp7b1 generating CA derivatives, 7α-hydroxy-cholestenoic acid (7α-OH-CA) and 7α-hydroxy-4-oxo-cholestenoic acid (7α-OH-4-CA). Genetic deletion of Cyp27a1 is predicted to decrease endogenous CA levels, whereas the deletion of Cyp7b1 is predicted to accumulate CA. **b** Cyp27a1 (−/−) increased brain mAβ42/Aβ40 ratio compared to Cyp27a1 (+/+) by ~20 %. **c-d** Cyp7b1 (−/−) decreased the ratio between mAβ42 to mAβ40 by ~20 % compared to Cyp7b1 (+/+), whereas increased the ratio of mAβ38/Aβ40 by ~30 %. 6–8 mice were tested in the group. The results were analyzed by one-way analysis of variance (ANOVA) followed by Dunnett’s multiple comparisons. *p < 0.05

To determine whether CA levels could affect Aβ42 production *in vivo*, we assessed Aβ levels in both Cyp27a1+/+, Cyp27a1+/− and Cyp27a1−/− mice brains and Cyp7b1+/+, Cyp7b1+/− and Cyp7b1−/− mice brains. In the brain, Cyp27a1 catalyzes the synthesis of CA from 27-OHC and Cyp7b1 catabolizes CA (Fig. 2a). From the previous literature, loss of Cyp27a1 has been shown to eliminate the levels of 27-OHC production in the mouse brain, suggesting decreased CA levels, whereas loss of Cyp7b1 increases mouse brain CA levels from ~30 nM to ~300 nM [36]. In humans with loss of function mutations in *CYP27A1* and *CYP7B1,* there is reduced and elevated CSF or plasma CA, respectively [36]. For our studies, mouse brains were harvested at 3 months and endogenous mouse Aβ levels measured by ELISA. Fig. 2b shows mAβ42/mAβ40 ratio measured from the Cyp27a1 mouse brains. There was a significant 23 % increase in the mAβ42/mAβ40 ratio in Cyp27a1−/− mice compared to Cyp27a1+/+ (Fig. 2b). Because Cyp27a1 mice were poor breeders, we did not obtain enough animals for an accurate measurement of brain Aβ38 levels. Conversely, Cyp7b1−/− mice showed a significant 21 % decrease in mAβ42/mAβ40 ratio (Fig. 2c) and a significant 25 % increase in mAβ38/mAβ40 ratio (Fig. 2d) compared to control Cyp7b1+/+ mice. In all cases, the ratios of mAβ40/mAβ42 and mAβ38/mAβ40 in heterozygous Cyp27a1 or Cyp7b1 mice (Cyp27a1+/− or Cyp7b1+/−) were intermediate between wild type and null animals, although the differences were not statistically significant. We attempted to observe amyloid plaque pathology in the context of Cyp7b1 or Cyp27a1 deficiency. Extensive efforts were made to breed APP_(KM670/671NL+V717F)_ CRND8 transgenic mice onto or Cyp7b1−/− genotype, but these efforts were unsuccessful. Though a few APP+/− on the Cyp7b1−/− backgrounds were generated, none of these survived past 3 months of age. Thus, we were unable to evaluate the effects of loss of Cyp7b1 on amyloid deposition.

### Intraperitoneal injections of CA did not alter mouse brain Aβ levels

**Fig. 3:**
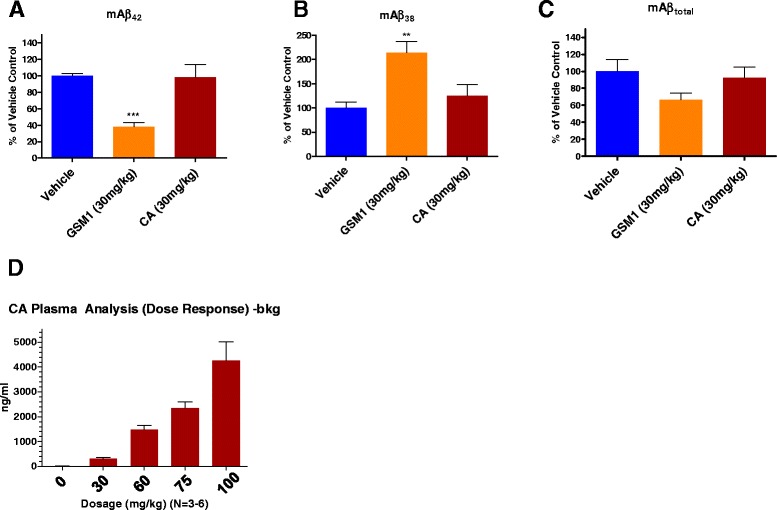
mAβ levels in the brains are measured after intraperitoneal (IP) injections of racemic mixture of CA in C57BL/6 and CF-1 mice (wild-type mice). At varying doses and time-points, CA did not show acute effects on brain mAβ42, mAβ38, and total mAβ levels. CA was administered at 30 mg/kg, 60 mg/kg, 75 mg/kg and 100 mg/kg and the brains were harvest 30 min after the injections in C57BL/6 or CF-1 mice. In addition, this was performed in time-course manner at 30 mg/kg. The brains were harvested at different time points (30 min, 1 h, 2 h and 3 h). The representative results of IP injections of GSM-1 and CA at 30 mg/kg at 1-h time point for mAβ42 (**a**), mAβ38 (**b**), and total Aβ (**c**) are demonstrated by mouse Aβ ELISAs. Solutol-based vehicle is utilized as a control and 6 mice were tested per group. (**d**) CA levels in the plasma in the wild type mice (*n* = 3-6) after IP injections at multiple doses are demonstrated. With the gradual increase in dosage (30 mg/kg, 60 mg/kg, 75 mg/kg, and 100 mg/kg), the plasma CA levels have increased. The results were analyzed by one-way analysis of variance (ANOVA) followed by Dunnett’s multiple comparisons. (***p < 0.001, **p < 0.01)

To test the acute effect of CA *in vivo*, CA (30 mg/kg) or GSM-1 (30 mg/kg), a potent GSM compound (Fig. 3), was given to wild type mice, and the brains were harvested after 30 min, 1, 2, and 3 h. While the positive control GSM-1 showed the expected Aβ modulation in mouse brains after three hours post intraperitoneal (IP) injection [37], there were no changes in Aβ levels after CA administration (Fig. 3a-c). It should be noted in these studies that the half-life (T_1/2_) for CA in humans is reported to be 90 min [38]. Next, a dose–response study was performed with CA given to wild type mice at 30, 60, 75, and 100 mg/kg doses with brains harvested 3 h after dosing. CA was well tolerated in mice up to the highest concentration (100 mg/kg). The Fig. 3d demonstrates CA plasma concentrations after injections of various doses in C57BL/6 mice. The standard curve for CA measurement showed a linear response with the limit of quantification of 100 ng/mL (~250 nM), which is close to physiological plasma CA concentration (100-300 nM) [39, 36, 40]. When different doses (30, 60, 75, 100 mg/kg) of CA were injected, plasma CA concentrations increased in dose dependent manner from 200 ng/mL (~500 nM) at 30 mg/kg dose to 4000 ng/mL (~10 μM) at 100 kg/mg dose (Fig. 3d). This might indicate some non-linearity in pharmacokinetics of CA. No significant effects of CA on mAβ42, mAβ38, and total mAβ compared to the vehicle-injected control were observed up to the highest 100 mg/kg dose (data not shown).

### GSM activity of CA analogs

**Fig. 4:**
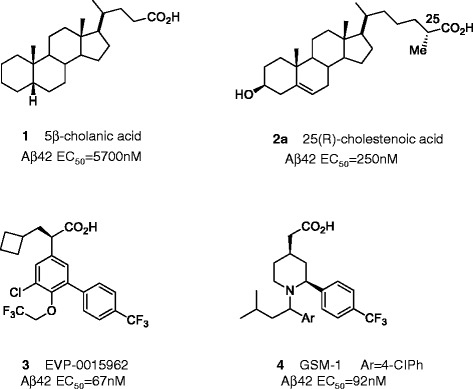
Chemical structures of acidic steroid type- and phenylacetic acid (PAA) type-GSMs with their EC_50_ for decreasing Aβ42. 5β-Cholanic acid (**1**) has a four-ring structure with a 4-carbon side chain on carbon 17 and its EC_50_ for decreasing Aβ42 is ~5.7 μM. 25(R)-cholesteonic acid (**2a**) has the same four-ring structure with an additional hydroxyl group on carbon 3 and a 6-carbon side chain on carbon 17. For this molecule, the EC_50_ is at ~250 nM. EVP-0015962 (**3**), (R)-2-(5-chloro-6-(2,2,2-trifluoroethoxy)-4’-(trifluoromethyl)biphenyl-3-yl)-3-cyclobutylpropanoic acid, shows GSM activity at EC_50_ of 67 nM from the previous literature. GSM-1 (**4**) has the two phenyl rings with the carboxylic acid functional group. The EC_50_ for GSM-1 is at 92 nM

We had previously screened 170 commercially available steroids and identified 5β-cholanic acid as the most potent steroid GSM within that set of compounds [26]. 5β-Cholanic acid **1** (Fig. 4) decreased Aβ42 with an EC_50_ of 5.7 μM [26]. In this report, we have identified CA **2a** as a potent GSM with an EC_50_ of 250 nM for decreasing Aβ42. As such, **2a** was comparable in potency to an optimized GSM clinical candidate phenylacetic acid EVP-0962 **3** and to preclinical tool compound GSM-1 **4**, as representative reference compounds from the carboxylic acid chemotype (Fig. 4).

A striking structural feature of CA relative to previous potent GSMs is the extended C5 alkylene tether linking the carboxylate group to the lipophilic core steroid nucleus. To date, potent GSMs such as **3** and **4** have been acetic acids in which the carboxylate group is linked to a core lipophilic moiety by a single carbon atom. To evaluate the effects of the alkylene tether, the structural-activity relationship (SAR) for CA analogs varying the tether from 3 methylenes to 7 methylenes (C3-C7) was examined (Table 1). The synthetic chemistry processes for both 25-(R) and 25-(S) pure diastereomers of CA, as well as the other CA analogs in Table 1, are illustrated and detailed in Additional file 1. Shorter tether analogs **5** (C3 tether analog) and **6** (C4 tether analog) displayed an order of magnitude lowering of potency with EC_50_ values of ~ 2.0 μM. The extended C6 and C7 tether analogs **10** and **11** exhibited a slight decline in potency (EC_50_ = 391 nM and 513 nM respectively) relative to the baseline C5 analog **7**.

**Table 1:** CA analogs EC_50_ for lowering Aβ42


R_1_	Cmpd#	Aβ42 EC_50_ (nM)
	5	1780
	6	1960
	7	110
	8	148
	2a	250
	2b	501
	9	2193
	10	391
	11	513

EC_50_=half maximal effective concentration

The SAR around the optimal C5 tether structure was elucidated with the analogs **2a, 2b, 7, 8** and **9**. The 25-(S)-CA, the methyl group diastereomer **2b**, was about 2-fold less potent than the 25-(R)-CA **2a**. The simple unsubstituted C5 methylene tether analog **7** displaying an EC_50_ of ~110 nM was more potent than **2a**. The corresponding α,β-unstaturated analog **8** was virtually equipotent to **7**. This steroid SAR at the α-carbon to the carboxylic acid group differs markedly from the SAR of PAA GSMs such as compound **3** (Fig. 4) where methyl substitution increases potency. Difluoro analog **9** prepared to favor the putative active ionized carboxylate, in fact, exhibited an order of magnitude loss in potency relative to the other C5 analogs.

**Fig. 5:**
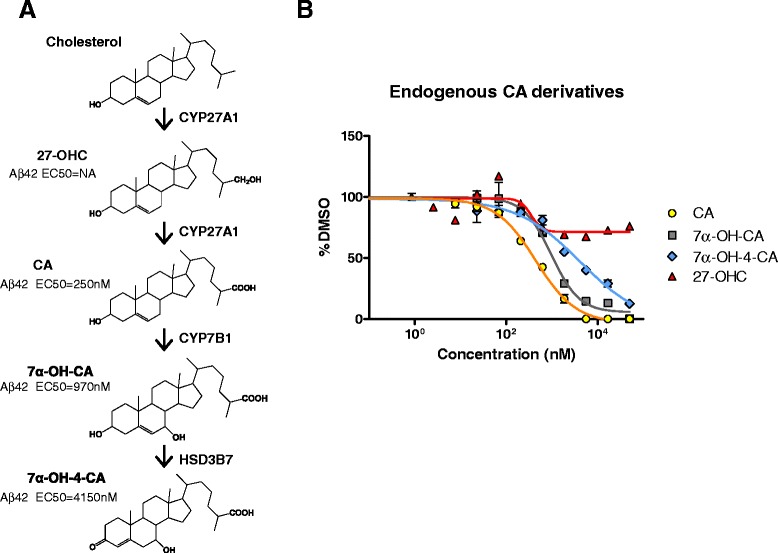
CA derivatives produced in the cholesterol elimination pathway decrease Aβ42 levels in CHO-2B7 cells. **a** Acidic pathway of the cholesterol elimination pathways is illustrated. Sequential hydroxylation on the carbon-23 by Cyp27a1 converts cholesterol to CA, and then Cyp7b1 converts CA to a CA derivative, 7α-OH-CA. 7α-OH-CA is further dehydrogenized by HSD3b7 producing 7α-OH-4-CA. **b** Dose–response curves were established to determine EC_50_ for the cholesterol metabolites found in this pathway using the conditioned media from CHO-2B7 cells treated with them. Compared to the DMSO control, CA and its derivatives showed Aβ decreasing effects at different EC_50_s. CA EC_50_ was at 250nM, 7α-OH-CA was at 970nM, and 7α-OH-4-CA was at 4150nM. 27-OHC EC_50_ could not be determined

We further explored the SAR of endogenous CA catabolites found in the acidic pathway (Fig. 5a). 27-OHC, the precursor of CA, is inactive as a GSM since it is missing the critical carboxylate group. CA is then converted to 7α-OH-CA and then to 7α-OH-3-CA, therefore we tested them for GSM activity in dose dependent studies (Fig. 5a). Both 7α-OH-CA and 7α-OH-3-CA demonstrated GSM activity but were not as potent as CA (Fig. 5a and b). Additionally, we aimed to substitute fluorine (F) at the carbon 3 and 7 positions of the CA catabolites as such fluorine substations can block metabolism (**12**–**13** in Table 2). Replacement of a 3-OH group of CA with a 3β-F group could be readily achieved to give **12** using standard methods. Somewhat surprisingly this modification in **12** resulted in great reduction in GSM potency. Attempts to synthesize the 7-F analog of CA were unsuccessful by routes based on standard diethylaminosulfur trifluoride (DAST) reaction of a corresponding 7-OH intermediate. 7-F delta-5-ene allyl fluoride steroid compounds could be isolated by DAST reaction, however these compounds demonstrated instability in our and previous studies [41, 42]. We also synthesized 3-deoxy-CA **13** based on our previous GSM SAR findings with cholenic acid analogs [26] and this demonstrated an EC50 of 670nM, approximately 3-fold less potent than CA. Collectively, these data demonstrate that endogenous **2b** CA is a relatively optimized steroid-GSM.

**Table 2:** Fluorine and deoxy CA analogs EC_50_ for lowering Aβ42


R_1_	R_2_	Cmpd #	Aβ42 EC_50_ (nM)
β-F	α-H	12	8906
β-H	β-H	13	671

EC_50_=half maximal effective concentration

### Carboxylic acid tether combined to PAA chemotypes did not show GSM activities

**Fig. 6:**
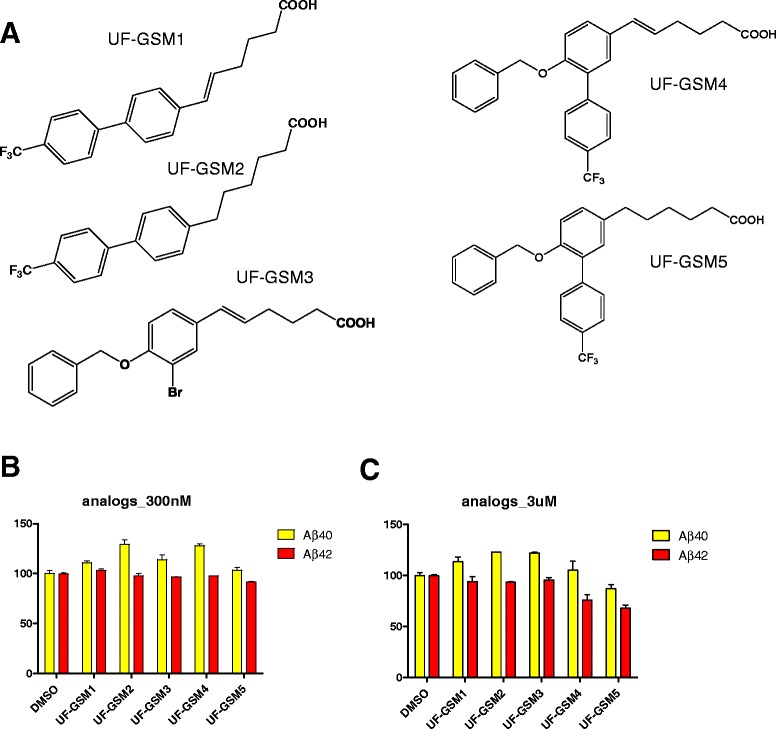
PAA-extended C5 alkylene tether grafting analogs. **a** PAA compounds combined with the COOH-tether were synthesized. **b**-**c** The treatments of the grafted compounds did not alter Aβ40 and Aβ42 levels significantly at 300nM or 3 μM concentrations

As phenyl acetic acid (PAA) chemotype GSMs have low nanomolar potencies for decreasing Aβ42 (Fig. 4, compound **3**), we examined whether increasing the length of the carboxylate tether to the PAA moiety could provide a path to further potency increases. The structures of the compounds synthesized are illustrated in Fig. 6a (the synthesis schemes are provided in Additional file 1). Biphenyl moieties (Fig. 6) were selected because they showed optimized drug potencies for GSM effects in previous studies [37, 43, 44]; however, no studies have been investigated regarding the PAA chemotype GSMs combined with the extended alkylene tether. Therefore, we decided to examine whether or not this feature can enhance potencies. We tested for potential GSM activity of these compounds by measuring Aβ42 levels at 300nM and 3 μM (Fig. 6b-c); however, these analogs did not demonstrate GSM activities at either concentration. Altogether, these data indicate that the increased potency observed with the C5 carboxylate tether appears to be specific to the steroid based GSMs and does not extend to other acid GSM chemotypes.

## Discussion

In this study, we identified CA as a potent acidic GSM with an EC_50_ for lowering Aβ42 of ~250 nM, a concentration well within the normal range of CA levels in human plasma (~100-300 nM). This data raised the possibility that CA was an endogenous GSM and that increasing brain CA levels might be a safe approach to lower brain Aβ42 levels. Peripheral dosing of CA, however, did not lower brain Aβ42 despite extremely high CA levels in the plasma (~10 μM), indicating that either CA does appear to readily cross the blood brain barrier or, if it does, is rapidly exported from the brain. Unfortunately, using our methodology, we were not able to accurately measure CA levels with sufficient sensitivity to accurately measure CA levels in the brains of these mice.

Given the potency of CA as a GSM, we explored whether mice with genetic deletions of Cyp27a1 [33, 34] and Cyp7b1 [35], the two enzymes regulating CA levels in the brain [31], showed alterations in mAβ42 levels. We found that the mAβ42/mAβ40 ratio was increased in the Cyp27a1−/− mice and mAβ42/mAβ40 ratio was decreased in Cyp7b1−/− mice, where CA levels were shown to decrease or increase CA levels, respectively [36]. Given that these shifts in ratio in these knockout mice are precisely what would be predicted if CA demonstrated GSM activity, we concluded that CA is likely to be a bona fide endogenous GSM synthesized in a cholesterol elimination pathway in brain [31]. Given the challenges of measuring levels of endogenous Aβ outside of the brain in wild type mice, we attempted to generate Cyp7b1−/−, APP+/− (CRND8) mice. Despite extensive efforts, we were unable to generate mice with this genotype that lived past 3 months. We did not attempt to cross the CRND8 mice with Cyp27a1−/− mice, because Cyp27a1−/− mice were even less fecund than the Cyp7b1−/− mice. Future studies in humans with genetic loss of function of *CYP27A1* that causes cerebrotendinous xanthomatosis (CTX) [45, 36] or with genetic loss of function of *CYP7B1* deficiency that causes liver failure in children or spastic paraplegia 5 (SPG5) in adults [46, 39, 36], might help to further establish the likelihood that CA is an endogenous GSM, as these patients show altered CA levels and would be predicted to have altered Aβ42/Aβ40 ratios [39, 36]; however, due to the small number of patients with these rare disorders, and the severe disease induced by loss of these CYP enzymes, such studies may be challenging to sufficiently power and control.

Building off our previous studies to examine a large number of steroids for GSM and inverse GSM (iGSM) activity [26], we synthesized a number of analogs to see if we can further increase potency. From these studies, we can conclude that CA seems to represent a relatively optimized steroid GSM, especially the C5 alkene tether linking the carboxylate group to the steroid backbone which appears to be optimal in length for maximizing steroid GSM potency. Indeed, there was a significant increase in GSM potency upon increasing the tether length from C3 to C5, but there was only a moderate loss of potency for increased C6 and C7 tether. Based on the observations from extended CA tether analogs, we explored the effects of C5 alkene tether carboxylates on other acidic GSM “scaffolds”. In all cases examined, this “grafting” approach decreased potency, indicating that the positon of the carboxylate group for optimal GSM potency is dependent on the overall structure of the molecule. Further modifications along the steroid backbone all decreased GSM activity relative to CA. For example, both endogenous CA metabolites 7α-OH-CA and 7α-OH-3-CA maintained GSM activity, but were less potent than CA.

Our findings that CA and other steroids can modify Aβ production expand the growing number of studies that demonstrate how cholesterol and other steroids can modulate Aβ profiles [47–54]. Of particular interest are studies showing that cholesterol binds to APP CTFβ [22, 23], albeit with low affinity, as this might suggest that CA, a cholesterol metabolite, could also interact with CTFβ. Our studies also show that CA behaves much like classic acidic GSMs and like all GSMs, exhibit a fairly flat SAR. Moreover, membrane lipids have been shown to alter the profile of Aβ produced [55], and therefore it is theoretically possible that CA could alter γ-secretase in a similar manner. However, given the nanomolar potency of CA and the aforementioned flat SAR, we think that this mechanism of action is unlikely. As our data show that it is challenging to generate CA analogs that retain potency, we have not attempted to generate CA analogs that could be used for affinity studies to identify primary binding sites. Given the nanomolar potency of CA, we speculate that it almost certainly interacts with PSEN/γ-secretase. However, as we have previously hypothesized, we would propose that most GSMs alter γ-secretase through a complex interaction involving both substrate and γ-secretase and possibly even other lipid membrane components [56, 57, 26, 58, 59]. Such a model is consistent with data showing that GSM effects are extremely sensitive to mutations within the substrate [59, 58, 60] and could explain why different GSM affinity probes have been shown to bind PSEN, PEN2 or C99 [56, 61–63]. It is important to consider that demonstrating binding with such a probe to a certain component does not rule out interaction with the other components, due to limitations where the reactive groups can be placed on the GSM affinity probes and the requirement for photoaffinity probes to have its photoaffinity label be in very close proximity to the bound protein.

In summary, although the endogenous metabolite CA is a potent γ-secretase modulator, i) its lack of ability to lower brain Aβ42 following peripheral dosing and ii) the inability to identify additional endogenous CA analogs with increased potency, suggests that pursuing CA or CA analogs for further preclinical development is not likely to be fruitful. Recent data show that CA can be toxic to primary mouse motor neuron in cultures [36] and raises concerns for pursuing CA or CA derivate as possible new small molecule therapeutics for AD. As the immediate precursor of CA, 27-OHC cholesterol, readily crosses the blood brain barrier, a pro-drug approach using a modified 27-OHC might be considered as an alternative strategy; however, emerging data that elevated 27-OHC may be a risk factor for osteoporosis and breast cancer, raises concerns about a 27-OHC cholesterol prodrug strategy to increase CA levels as well [64, 65].

## Methods

### Cell culture and drug treatment

Chinese hamster ovary (CHO) cells stably overexpressing APP695 (CHO-2B7 cells) [66] were grown in Ham’s F-12 medium (Life Technologies) supplemented with 10 % fetal bovine serum and 100 units/ml of penicillin and 100 μg/ml streptomycin. Cells were grown at 37 °C in a humidified atmosphere containing 5 % CO_2_ in tissue culture plates (Costar). The cells were harvested at confluence and then utilized for biochemical analyses. Compounds were dissolved in dimethyl sulfoxide (DMSO) and screened in CHO-2B7 cells. The cells were incubated for 16 h in the presence of the compound diluted into OptiMEM-reduced serum medium (Life Technologies, Carlsbad, CA, USA) containing 1 % fetal bovine serum. Compounds used for our study were either purchased from Avanti Polar Lipids, Inc. or synthesized by SAI Life Sciences Ltd. The synthesis schemes of the newly synthesized compounds are demonstrated in Additional file 1.

### *In vitro* γ-secretase assay

Broken cell assays were performed with slight modifications from the previous studies [67, 18]. The membrane derived from the H4 neuroglioma cells overexpressing APP695wt were prepared by carbonate extraction and incubated at 37 °C for 2 h with CA at various concentrations. Aβ levels were quantified by sandwich ELISAs. For Aβ and AICD spectra, the recombinant C100Flag proteins were overexpressed and purified from *Escherichia coli* BL21 using a HiTrap Q-column (GE Life Science, Little Chalfont, U.K.) [68, 69, 58]. The membrane containing γ-secretase was isolated from the CHO S-1 cell line using sodium carbonate (100 mM, pH 11.0) [70]. For the *in vitro* γ-secretase assay, C100Flag recombinant protein at 25 μM was incubated with the membrane (100 μg/mL) in the presence of CA (20 μM) and DMSO in 150 mM sodium citrate buffer (pH 6.8) containing complete protease inhibitor (Roche, Indianapolis, IN) for 2 h at 37 °C.

### Mice

All procedures were performed according to the National Institute of Health Guide for the Care and Use of Experimental Animals and were approved by the University of Florida Institutional Animal Care and Use Committee. The Cyp27a1−/− (B6.129-Cyp27a1tm1Elt/J) and Cyp7b1−/− (B6;129S-Cyp7b1tmRus/J) strains were obtained from Jackson Laboratory (Bar Harbor, ME). Cyp27a1−/− mice were bred with C57BL/6 in order to produce the heterozygous littermates of Cyp27a1, and Cyp7b1−/− mice were bred with C57BL/6 mice to produce the heterozygous Cyp7b1 littermates. The wild type, heterozygous, and knockout littermates of Cyp27a1 and Cyp7b1 mice were generated from Cyp27a1+/− X Cyp27a1+/− and Cyp7b1+/− X Cyp7b1+/−, respectively.

### Primary mixed neuron-glia culture

Primary mixed neuron-glia cultures were prepared from postnatal day 0 (P0) C3HBL/6 mouse brains (Harlan Labs). Cerebral cortices were dissected from P0 mouse brains and were dissociated in 2 mg/ml papain (Worthington) and 50 μg/mL DNAse I (Sigma) at 37 °C for 20 min. They were then washed three times in sterile Hank’s balanced salt solution (HBSS) to inactivate the papain and switched to 5 % fetal bovine serume (HyClone) in Neurobasal-A growth media (Gibco), which includes 0.5 mM L-glutamine (Gibco), 0.5 mM GlutaMax (Life Technologies), 0.01 % antibiotic-antimycotic (Gibco), and 0.02 % SM1 supplement (Stemcell). The tissue mixture was then triturated three times using a 5 mL pipette followed by a Pasteur pipette, and strained through a 70 μm cell strainer. The cell mixture was then centrifuged at 200xg for 3 min, and re-suspended in fresh Neurobasal-A media. They were then plated onto poly-D-lysine coated 96well plates at 100,000 cells/well. Cells were maintained in the Neurobasal-A growth media mentioned above without fetal bovine serum (FBS) at 37 °C in a humidified 5 % CO_2_ chamber.

### CA IP injections

25(R)-CA powder was initially dissolved in DMSO (<4.5 % in the final mixture) and then combined with polyethylene glycol (15)-hydroxystearate (Solutol), ethanol, and water at a ratio of (15:10:75). One molar equivalent of sodium hydroxide was added to the mixture [71, 72]. We performed CA intraperitoneal (IP) injections to wild-type mice (C57BL/6 or CF-1). The mice were injected with 25(R)-CA on the right side of the abdomen. The injections have been performed with various time points (30 min, 1 h, 2 h, and 3 h) and with multiple doses (30 mg per kg (mg/kg), 60 mg/kg, 75 mg/kg and 100 mg/kg). The number of each cohort is 6–8. We used 30 mg/kg of CA for the time-course experiments, and for the dose–response experiments the end-point was set at 30 min. The brains and serum are harvested and frozen for brain Aβ extraction.

### Brain Aβ extraction

The mouse brains were harvested at the age of 3 months. The brains were weighed and recorded. The Diethylamine/Sodium Chloride (DEA/NaCl) extraction buffer (0.4 % DEA) was added to each sample and homogenized using a sonicator. The samples were transferred to a poly-carbonate centrifuge tube and spun down at 50,000xg for 30 min at 4 °C. The supernatant was loaded on the vacuum manifold with the appropriate number of HLB Oasis columns. The samples were loaded on the conditioned column, filtered, and eluted using prepared elution buffer (90 % Methanol, 2 % NH4OH). The eluates are concentrated using the Thermo-Savant SpeedVac concentrator for a minimum of 2 h at 55 °C with radiant heat. The concentrated samples are reconstituted in a blocking buffer (0.67 % Bovine serum albumin (BSA)) at the appropriate volume.

### Plasma CA analysis

The plasma samples were extracted using published solid phase extraction method (72) and analyzed by HPLC-MS-MS. Briefly, 0.1 ml mouse plasma samples after adding 20 μl of D3-CA as internal standard were preconditioned with 1.4 ml of ethanol (99.9 %), and 0.5 ml of water, centrifuged at 4 °C, 4000 rpm for 10 min. This solution was then loaded onto a Sep-Pak tC18 (SPE1) solid phase extraction cartridge which were preconditioned with 70 % ethanol. The sample was washed with one column volume of 70 % ethanol then eluted from the column by 2 + 1 ml of 99.9 % ethanol; it was dried in centrifuge evaporator. The residue was reconstituted in 100 μl of isopropanol. It was oxidized by adding 1 ml of 50 mM phosphate buffer (pH = 7) containing 3 μl of cholesterol oxidase and incubated at 37 °C for 1 h, quenched with 1.9 ml of methanol. The mixture was further processed by adding 150 μl glacial acetic acid and 1 smidgen (about 80 mg) GP reagent {1-(carboxymethyl) pyridinium chloride hydrazide} and incubated at room temperature overnight in the dark. On the next day, a second solid phase extraction [73] was employed to separate the derivatized CA from the excess derivatization reagent using the following: Sep-pak C18 (SPE 2, different from SPE1) cartridge with 1 column volume of 99.9 % methanol and 1 column volume of 10 % methanol, after application of the sample wash with 10 % methanol, then elute with 2*1 ml of 100 % methanol. Mix 200 μl of the elution solution with 50 μl of water to obtain 250 μl of 80/20 (methanol/water, v/v) samples. 20 μl was injected onto HPLC-MS-MS for analysis.

HPLC-MS-MS conditions: HPLC contains a Perkin Elmer series 200 autosampler and a Perkin Elmer series 200 pump, MS-MS was Waters Quattro LC-Z, ES positive mode, Cone voltage 45 volts, collision energy 30volts, Desolvation temperature 350 °C. Source block temperature 120 °C. MS/MS transitions: CA 549.0/470.0; D3-CA 552.0/473.0. HPLC mobile phase was 80/20 Methanol/water(v/v) containing 0.1 %Formic Acid, HPLC column was ThermoFisher Hypersil Gold, 50*2.1 mm, 1.9 μ, flow rate 0.2 ml/min. Injection volume 20 μl, run time 4 min, CA retention time 1.4 min.

### Antibodies and ELISAs

Monoclonal antibodies to Aβ were generated by the Mayo Clinic Immunology Core facilities (Jacksonville, FL, USA). Ab5 recognizes an epitope in the amino terminus of Aβ (Aβ1-16), recognizes both monomeric and aggregated Aβ, and is human specific. Ab13.1.1. was raised against Aβ35-40 and is specific for Aβx-40, and exhibits minimal cross-reactivity with other Aβ peptides. Ab 2.1.3 was raised against Aβ35-42 and is specific for Aβx-42. The Aβ38 antibody (Ab38), supplied by P. Mehta (Institute of Basic Research, Staten Island, NY, USA), specifically recognizes Aβx-38 and shows no cross-reactivity with other Aβ peptides [74]. For cell-based screens, Aβ was captured from conditioned medium with either Ab5, Ab38, Ab13.1.1, or Ab2.1.3 (coated at 10-50 μg/ml in EC buffer: 5 mM NaH2PO4-H2O, 20 mM Na2HPO4, 400 mM NaCl, 2.5 mM EDTA-full name, 151.5 μM BSA, 813 μM CHAPS, and 7.7 mM NaN3) on Immulon 4HBX Flat-Bottom Microfilter 96-well plates (Thermo Scientific, Waltham, MA, USA). Total Aβ level was determined by capture with Ab5 and detected with horseradish peroxidase (HRP)-conjugated 4G8 (a monoclonal antibody against Aβ17-24; Covance, Waltham, MA, USA) with the other Aβ peptides detected with HRP-conjugated Ab5. For the cell-free assay and measuring mouse endogenous Aβ, HRP-conjugated 4G8 was used as the secondary detection antibody. Aβ standards (Bachem, King of Prussia, PA, USA) were prepared by dissolving in hexafluoroisopropanol (HFIP) at 1 mg/ml with sonication, dried under nitrogen, resuspended at 2 mg/ml HFIP, sonicated again and dried under nitrogen. The resulting Aβ was resuspended in 0.01 % ammonium hydroxide, portioned into aliquots in EC buffer, and frozen at −80 °C. Following these steps, the Aβ is monomeric, as determined by size-exclusion chromatography.

### Immunoprecipitation-Mass spectrometry

Conditioned media from the CHO-2B7 cells and the samples prepared from *in vitro* γ-secretase studies were used to analyze Aβ and AICD profiles using matrix-assisted laser desorption/ionization time of flight (MALDI-TOF) mass spectrometry analysis. The secreted Aβ peptides were analyzed as previously described with the following modifications [2, 75, 76]. Briefly, the Aβ peptides were immunoprecipitated using Ab5 recognizing the Aβ1-16 epitope [77] and sheep anti-mouse IgG magnetic Dynabeads (Life Technologies, catalog no. 11201D) and the AICD fragments were captured using anti-Flag M2 magnetic beads (Sigma). The samples were washed and eluted with 10 μM solution of 0.1 % trifluoroacetic acid (TFA) in water. Eluted samples were mixed 2:1 with saturated α-cyano-4-hydroxycinnamic acid (CHCA) matrix (Sigma) in acetonitrile: methanol (60:40 %) and loaded onto a CHCA pretreated MSP 96 target plate-polished steel (Bruker, Billerica, MA, USA - Part No.224989). Samples were analyzed using a Bruker Microflex LRF-MALDI-TOF mass spectrometer.

### Statistics

*In vitro* data were expressed and graphed as the mean ± SEM using GraphPad Prism 5 software. Analysis was by one-way analysis of variance (ANOVA) followed by Dunnett’s multiple comparisons, and was by two-way analysis of variance (ANOVA) followed by bonferroni post-hoc testing for group differences. The level of significance was set at *p* < 0.05 in all tests.

## Additional file

Additional file 1:
**Schematics of syntheses of (25R)-cholestenoic acid and its analogs.**

## Abbreviations

Aβ: Amyloid-β
AD: Alzheimer’s disease
APP: Amyloid precursor protein
AICD: APP intracellular domain
CTF: APP carboxyl terminal fragment
CA: Cholestenoic acid
Cyp: Cytochrome P450
CHO: Chinese hamster ovary
CTX: Cerebrotendinous xanthomatosis
GSM: γ-secretase modulator
NSAID: Non-steroidal anti-inflammatory drug
SPG5: Spastic paraplegia 5
SAR: Structure-activity relationship
