# Supplementary material for: Cholestenoic acid, an endogenous cholesterol metabolite, is a potent γ-secretase modulator
Source: Mol Neurodegener. 2015 Jul 14;10:29. doi: 10.1186/s13024-015-0021-z (PMC4501119; doi:10.1186/s13024-015-0021-z)
Supplement: Additional file 1: — Schematics of syntheses of (25R)-cholestenoic acid and its analogs. [file 13024_2015_21_MOESM1_ESM.pdf]

Synthesis of (25*R*)-Cholestenoic acid

Scheme 1:

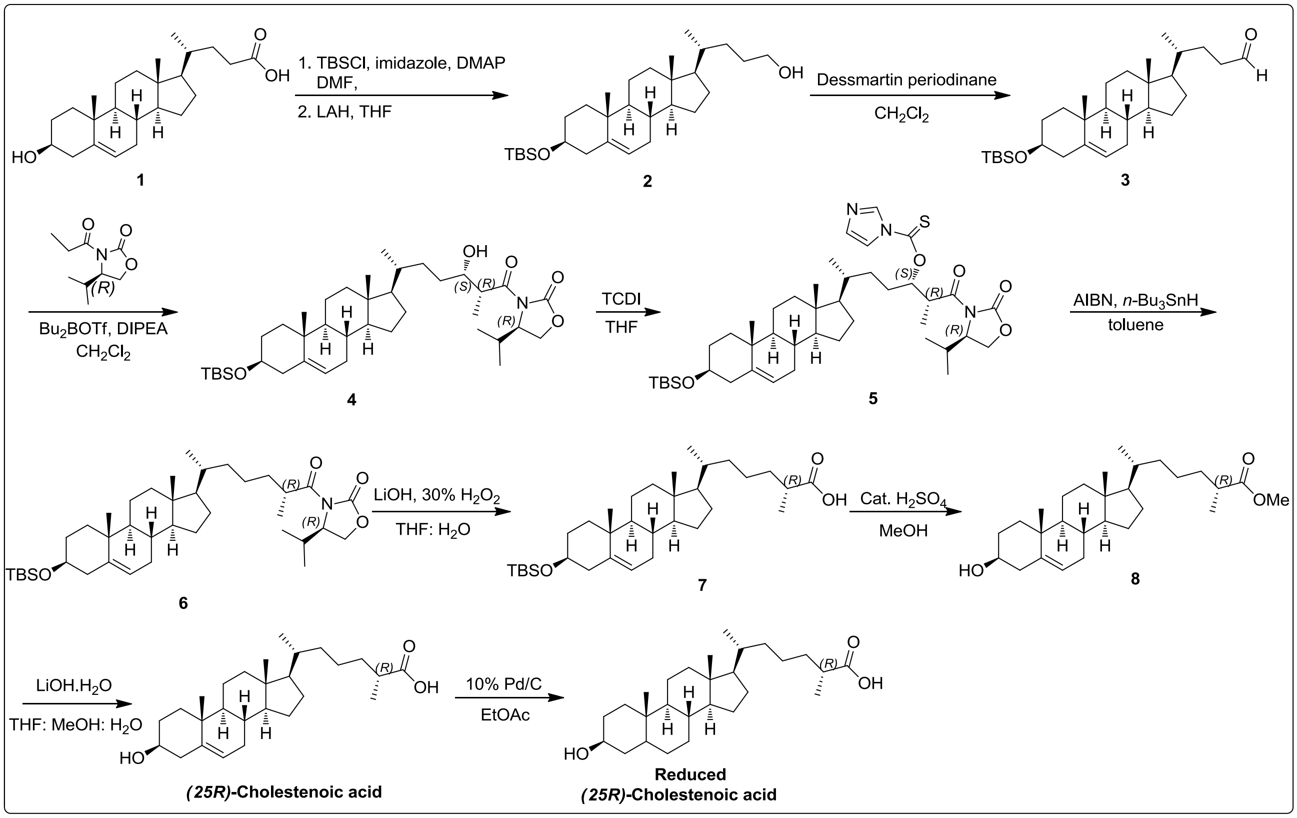

Experimental:

Synthesis of 3β-(*tert*-Butyldimethylsilyloxy) chol-5-en-24-ol (2):

To a stirred solution of 3β-hydroxy chol-5-ene-24-oic acid **1** (5.0 g, 13.35 mmol) in DMF (75 mL) under inert atmosphere were added Imidazole (7.2 g, 106 mmol) and 4-Dimethyl amino pyridine (3.5 g, 29 mmol) at RT and stirred for 10 min. Then TBS-Cl (6.4 g, 43 mmol) was added to the reaction mixture at 0 ^oC; warmed to RT and stirred for 16 h. The progress of the reaction was monitored by TLC; after completion of the reaction, the reaction mixture was diluted with ice cold water (50 mL) and extracted with *n*-Hexane (2 x 50 mL). The combined organic extracts were washed with water (50 mL), dried over sodium sulphate, filtered and concentrated under reduced pressure to obtain 10 g of diTBS protected compound. The diTBS protected compound (1 g, 1.65 mmol) was dissolved in THF (7 mL) was added to the solution of Lithium aluminum hydride (63 mg, 1.65 mmol) in THF (3 mL) at 0 ^oC; warmed to RT and stirred for 16 h. The progress of the reaction was monitored by TLC; after completion of the reaction, the reaction mixture was quenched with saturated aqueous sodium sulphate, and the compound was extracted with EtOAc (2 x 25 mL). The combined organic extracts were washed with water (20

mL), brine (20 mL), dried over sodium sulphate, filtered and concentrated under reduced pressure to obtain the crude. The crude was purified through flash chromatography on silica gel using 5% EtOAc/*n*-Hexane to afford compound **2** (266 mg, 33%) as white solid.

^{1}H NMR (400 MHz, $CDCl_3$): δ 0.05 (s, 6H), 0.67 (s, 3H), 0.88 (s, 9H), 0.93 (d, *J* = 6.6 Hz, 3H), 0.95-1.17 (m, 6H), 0.98 (s, 3H), 1.21-1.27 (m, 2H), 1.39-1.72 (m, 11H), 1.79 (dt, *J* = 13.3, 3.5 Hz, 1H), 1.82-1.84 (m, 1H), 1.93-2.01 (m, 2H), 2.15 (ddd, *J* = 13.3, 4.9, 2.2 Hz, 1H), 2.24-2.26 (m, 1H), 3.45-3.50 (m, 1H), 3.60-3.62 (m, 2H), 5.30-5.31 (m, 1H).

3β-(*tert*-Butyldimethylsilyloxy) chol-5-en-24-al (3):

To a stirred solution of compound **2** (2.0 g, 4.21 mmol) in CH_2Cl_2 (20 mL) under inert atmosphere was added Dess-martin periodinane (5.3 g, 12.65 mmol) at 0 ^oC; warmed to RT and stirred for 2 h. The progress of the reaction was monitored by TLC; after completion of the reaction, the reaction mixture was diluted with water (30 mL) and extracted with CH_2Cl_2 (2 x 30 mL). The combined organic extracts were washed with water (30 mL), brine (30 mL), dried over sodium sulphate, filtered and concentrated under reduced pressure to obtain the crude. The crude compound was purified through flash chromatography on silica gel using 5% EtOAc/*n*-Hexane to afford compound **3** (1.1 g, 60%) as white solid.

^{1}H NMR (500 MHz, $CDCl_3$): δ 0.05 (s, 6H), 0.68 (s, 3H), 0.88 (s, 9H), 0.92 (d, *J* = 6.4 Hz, 3H), 0.99 (s, 3H), 1.05-1.19 (m, 4H), 1.25-1.40 (m, 3H), 1.43-1.52 (m, 5H), 1.55-1.61 (m, 1H), 1.70-1.85 (m, 4H), 1.94-2.00 (m, 2H), 2.26-2.45 (m, 4H), , 2.17-2.19 (m, 2H), 3.46-3.49 (m, 1H), 5.30-5.32 (m, 1H), 9.76 (s, 1H).

(4*R*, 24'*S*, 25'*R*)-3-[3'β-(*tert*-Butyldimethylsilyloxy)-24'-hydroxycholest-5'en-26'-oyl]-4-isopropyloxazolidin-2-one (4):

To a stirred solution of (R)-4-isopropyl-3-propionyloxazolidin-2-one (1.78 g, 9.63 mmol) in dry CH_2Cl_2 (20 mL) under inert atmosphere were added dibutyl boron triflate (2.9 g, 10.60 mmol) and diisopropyl ethyl amine (2.16 mL, 12.45 mmol) at 0 ^oC and stirred for 30 min. Then compound **3** (3.5 g, 7.4 mmol) in CH_2Cl_2 (20 mL) was added to the reaction mixture at -78 ^oC and stirred for 30 min; warmed to 0 ^oC and stirred for 80 min. Then methanol (14 mL) and 30% hydrogen peroxide (14 mL) were added to the reaction mixture at 0 ^oC; warmed to RT and stirred for 30 min. The progress of the reaction was monitored by TLC; after completion of the reaction, the volatiles were evaporated under reduced pressure. The residue was diluted with water (30 mL) and extracted with CH_2Cl_2 (2 x 30 mL). The combined organic extracts were washed with water (30 mL), dried over sodium sulphate, filtered and concentrated under

reduced pressure to obtain the crude. The crude was purified through flash chromatography on silica gel using 12 % EtOAc/*n*-Hexane to afford compound **4** (2.7 g, 56%) as white solid.

^{1}H NMR (400 MHz, $CDCl_3$): δ 0.05 (s, 6H), 0.64 (s, 3H), 0.87 (s, 12H), 0.87-0.99 (m, 13H), 1.11 (s, 3H), 1.14-1.25 (m, 4H), 1.43-1.44 (d, *J* = 6.0 Hz, 3H), 1.47-.1.52 (m, 7H), 1.72-1.77 (m, 1H), 1.78-1.81 (m, 2H), 1.93-2.01 (m, 2H), 2.17-2.18 (m, 1H), 2.33-2.36 (m, 2H), 3.02-3.03 (m, 1H), 3.45-3.50 (m, 1H), 3.76-3.78 (m 1H), 3.86-3.88 (m, 1H), 4.20-4.30 (m, 2H), 4.45-4.49 (m, 1H), 5.29-5.31 (m, 1H).

(4*R*, (24'*S*, 25'*R*)-3-[3'β-(*tert*-Butyldimethylsilyloxy)-24'-(*O*-thiocarbonyl imidazolyl) cholest-5'-en-26'-oyl]-4-isopropyloxazolidin-2-one (5):

To a stirred solution of compound **4** (500 mg, 0.75 mmol) in dry THF (10 mL) under inert atmosphere was added thiocarbonyl diimidazole (973 mg, 5.47 mmol) at RT; heated to reflux and stirred for 20 h. The progress of the reaction was monitored by TLC; after completion of the reaction, the volatiles were evaporated under reduced pressure to obtain the crude. The crude was purified through flash chromatography on silica gel using 15% EtOAc/*n*-Hexane to afford compound **5** (480 mg, 82%) as white solid.

^{1}H NMR (400 MHz, $CDCl_3$): δ 0.05 (s, 6H), 0.64 (s, 3H), 0.87 (s, 9H), 0.87-0.99 (m, 17H), 1.11 (s, 3H), 1.14-1.25 (m, 3H), 1.43-1.44 (d, *J* = 6.0 Hz, 3H), 1.47-.1.52 (m, 7H), 1.72-1.77 (m, 1H), 1.78-1.81 (m, 2H), 1.93-2.01 (m, 2H), 2.17-2.18 (m, 1H), 2.33-2.36 (m, 2H), 3.46-3.48 (m, 1H), 4.21-4.30 (m, 4H), 5.30-5.31 (m, 1H), 6.01-6.03 (m 1H), 7.03-7.04 (m, 1H), 7.59-7.60 (m, 1H), 8.31-8.30 (m, 1H).

(4*R*, 25'*R*)-3-[3'β-(*tert*-Butyldimethylsilyloxy)-cholest-5'-en-26'-oyl]-4-isopropyl oxazolidin-2-one (6):

To a stirred solution of compound **5** (480 mg, 0.62 mmol) in toluene (5 mL) under inert atmosphere were added AIBN (10 mg, 0.06 mmol) and tributyl tin hydride (363 mg, 1.25 mmol) at RT; heated to reflux and stirred for 1 h. The progress of the reaction was monitored by TLC; after completion of the reaction, the volatiles were evaporated under reduced pressure to obtain the crude. The crude was purified through silica gel column chromatography using 5% EtOAc/ *n*-Hexane to afford compound **6** (270 mg, 67%) as white solid.

^{1}H NMR (400 MHz, $CDCl_3$): δ 0.05 (s, 6H), 0.64 (s, 3H), 0.87 (s, 12H), 0.87-0.99 (m, 13H), 1.11 (s, 3H), 1.14-1.25 (m, 4H), 1.43-1.44 (d, *J* = 6.0 Hz, 3H), 1.47-.1.52 (m, 9H), 1.72-1.77 (m, 1H), 1.78-1.81 (m, 2H), 1.93-2.01 (m, 2H), 2.17-2.18 (m, 1H), 2.33-2.36 (m, 2H), 3.45-3.50 (m, 1H), 3.70-3.73 (m, 1H), 4.18-4.33 (m, 2H), 4.44-4.47 (m, 1H), 5.29-5.31 (m, 1H).

(25*R*)-3β-(*tert*-Butyldimethylsilyloxy) cholest-5-en-26-oic Acid (7):

To a stirred solution of compound **6** (270 mg, 0.42 mmol) in THF: H_2O (3:1, 4 mL) were added 30% hydrogen peroxide (85 mg, 2.52 mmol) and lithium hydroxide monohydrate (35 mg, 0.84 mmol) at 0 ^oC and stirred for 3 h. The progress of the reaction was monitored by TLC; after completion of the reaction, the reaction mixture was quenched with aqueous sodium sulfite solution (10 mL), acidified with 1 N HCl solution (10 mL) and extracted with diethyl ether (2 x 20 mL). The combined organic extracts were washed with water (10 mL), dried over sodium sulphate, filtered and concentrated under reduced pressure to obtain the crude. The crude was purified through silica gel column chromatography using 15% EtOAc/ *n*-Hexane to afford compound **7** (200 mg, 90%) as white solid.

^{1}H NMR (400 MHz, $CDCl_3$): δ 0.05 (s, 6H), 0.68 (s, 3H), 0.89 (s, 9H), 0.90 (d, *J* = 6.5 Hz, 3H), 1.01 (s, 3H), 1.02-1.21 (m, 5H), 1.26 (d, J = 6.6 Hz, 3H), 1.47-1.60 (m, 15H), 1.62-1.78 (m, 2H), 1.80-1.97 (m, 2H), 1.99-2.19 (m, 2H), 2.20-2.31 (m, 2H), 2.46-2.51 (m, 1H), 3.45-3.52 (m, 1H), 5.32-5.33 (m, 1H).

Methyl (25*R*)-3β-hydroxycholesten-5-en-26-oate (8):

To a stirred solution of compound **7** (150 mg, 0.28 mmol) in methanol (3 mL) under inert atmosphere was added concentrated sulphuric acid (catalytic amount) at 0 ^oC; heated to reflux and stirred for 16 h. The progress of the reaction was monitored by TLC; after completion of the reaction, the volatiles were evaporated under reduced pressure. The residue was diluted with ice cold water (10 mL) and extracted with CH_2Cl_2 (2 x 10 mL). The combined organic extracts were washed with saturated $NaHCO_3$ solution (10 mL), dried over sodium sulfate, filtered and concentrated under reduced pressure to obtain the crude. The crude was purified through silica gel column chromatography using 20% EtOAc/ *n*-Hexane to afford compound **8** (70 mg, 58%) as white solid.

^{1}H NMR (400 MHz, $CDCl_3$): δ 0.66 (s, 3H), 0.88-1.16 (m, 8H), 0.89 (d, *J* = 6.5 Hz, 3H), 0.99 (s, 3H), 1.17-1.66 (m, 15H), 1.76-1.86 (m, 4H), 1.94-2.02 (m, 2H), 2.22-2.31 (m, 2H), 2.40-2.46 (m, 1H), 3.48-3.52 (m, 1H), 3.66 (s, 3H), 5.34-5.35 (m, 1H).

(25*R*)-Cholestenoic acid [(25*R*)-3β-Hydroxycholest-5-en-26-oic Acid] :

To a stirred solution of **8** (300 mg, 0.69 mmol) in THF: MeOH: H_2O (2:1:1, 8 mL) was added lithium hydroxide monohydrate (94 mg, 2.23 mmol) at RT and stirred for 16 h. The progress of the reaction was monitored by TLC; after completion of the reaction, the volatiles were

evaporated under reduced pressure. The residue was diluted with water (20 mL), acidified with 1 N HCl solution (10 mL) and extracted with CH_2Cl_2 (2 x 20 mL). The combined organic layers were dried over sodium sulfate, filtered and concentrated under reduced pressure to obtain the crude. The crude was triturated with acetonitrile (2 x 4 mL) to afford **(25R)-Cholestenoic acid** (200 mg, 69%) as white solid.

^{1}H NMR (500 MHz, $CDCl_3$): δ 0.67 (s, 3H), 0.87-1.27 (m, 9H), 0.9 (d, *J* = 6.5 Hz, 3H), 0.99 (s, 5H), 1.17 (d, *J* = 7.0 Hz, 3H), 1.31-.178 (m, 12H), 1.79-1.86 (m, 2H), 1.93-2.07 (m, 2H), 2.22-2.31 (m, 2H), 2.45-2.50 (m, 1H), 3.48-3.56 (m, 1H), 5.34-5.35 (m, 1H).

Synthesis of (25*S*)-Cholestenoic acid

Scheme 2:

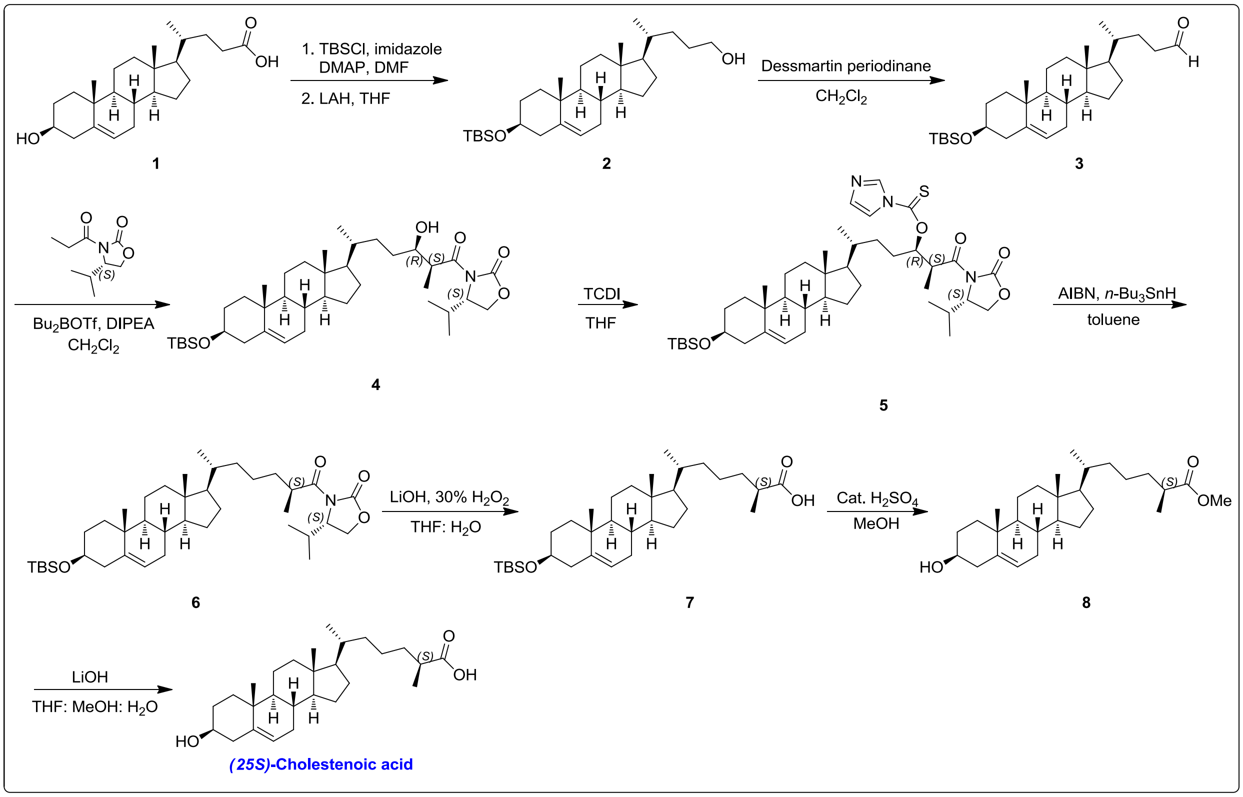

Experimental:

(4*S*, 24'*R*, 25'*S*)-3-[3'β-(*tert*-Butyldimethylsilyloxy)-24'-hydroxycholest-5'en-26-oyl]-4-isopropyloxazolidin-2-one (4):

To a stirred solution of (S)-4-isopropyl-3-propionyloxazolidin-2-one (2 g, 11.01 mmol) in dry CH_2Cl_2 (20 mL) under inert atmosphere were added dibutyl boron triflate (3.2 g, 12.00 mmol) and diisopropyl ethyl amine (2.46 mL, 14.11 mmol) at 0 ^oC and stirred for 30 min. Then compound **3** (4 g, 8.47 mmol) in CH_2Cl_2 (20 mL) was added to the reaction mixture at -78 ^oC and stirred for 30 min; warmed to 0 ^oC and stirred for 80 min. Then methanol (40 mL) and 30% hydrogen peroxide (40 mL) were added to the reaction mixture at 0 ^oC; warmed to RT and stirred for 30 min. The progress of the reaction was monitored by TLC; after completion of the reaction, the volatiles were evaporated under reduced pressure. The residue was diluted with water (30 mL) and extracted with CH_2Cl_2 (2 x 30 mL). The combined organic extracts were washed with water (30 mL), dried over sodium sulphate, filtered and concentrated under reduced pressure to obtain the crude. The crude was purified through silica gel column chromatography using 12% EtOAc/ *n*-Hexane to afford compound **4** (3 g, 54%) as white solid.

^{1}H NMR (400 MHz, $CDCl_3$): δ 0.05 (s, 6H), 0.67 (s, 3H), 0.88-0.92 (m, 21H), 1.04 (s, 4H), 1.07-1.26 (m, 3H), 1.24-1.26 (m, 6H), 1.39-1.48 (m, 6H), 1.58-1.61 (m, 2H), 1.69-1.87 (m, 3H), 1.94-2.01 (m, 2H), 2.14-2.37 (m, 3H), 2.89 (s, 1H), 3.45-3.50 (m, 1H), 3.87-3.89 (m, 2H), 4.20-4.30 (m, 2H), 4.46-4.48 (m, 1H), 5.30-5.31 (m, 1H).

(4*S*, (24'*R*, 25'*S*)-3-[3'β-(*tert*-Butyldimethylsilyloxy)-24'-(*O*-thiocarbonyl imidazolyl) cholest-5'-en-26'-oyl]-4-isopropyloxazolidin-2-one (5):

To a stirred solution of compound **4** (3 g, 4.55 mmol) in dry THF (30 mL) under inert atmosphere was added thiocarbonyl diimidazole (5.8 g, 32.82 mmol) at RT; heated to reflux and stirred for 20 h. The progress of the reaction was monitored by TLC; after completion of the reaction, the volatiles were evaporated under reduced pressure to obtain the crude. The crude was purified through silica gel column chromatography using 15% EtOAc/ *n*-Hexane to afford compound **5** (3 g, 85%) as white solid.

^{1}H NMR (400 MHz, $CDCl_3$): δ 0.05 (s, 6H), 0.65 (s, 3H), 0.86 (s, 9H), 0.87-0.92 (m, 13H), 0.99 (s, 3H), 1.08-1.25 (m, 4H), 1.31 (d, *J* = 6.8 Hz, 3H), 1.47-1.51 (m, 8H), 1.55-1.60 (m, 1H), 1.69-1.81 (m, 3H), 1.90-1.98 (m, 3H), 2.13-2.35 (m, 3H), 3.43-3.51 (m, 1H), 4.19-4.33 (m, 4H), 5.29-5.31 (m, 1H), 6.02-6.06 (m 1H), 7.04 (s, 1H), 7.60 (s, 1H), 8.31 (s, 1H).

(4*S*, 25'*S*)-3-[3'β-(*tert*-Butyldimethylsilyloxy)- cholest-5'-en-26'-oyl]-4-isopropyloxazolidin-2-one (6):

To a stirred solution of compound **5** (3 g, 3.90 mmol) in toluene (30 mL) under inert atmosphere were added AIBN (25 mg, 0.01 mmol) and tributyl tin hydride (2.27 g, 7.81 mmol) at RT; heated to reflux and stirred for 30 min. The progress of the reaction was monitored by TLC; after completion of the reaction, the volatiles were evaporated under reduced pressure to obtain the crude. The crude was purified through silica gel column chromatography using 3% EtOAc/ *n*-Hexane to afford compound **6** (1.8 g, 72%) as white solid.

^{1}H NMR (400 MHz, $CDCl_3$): δ 0.05 (s, 6H), 0.66 (s, 3H), 0.86 (s, 9H), 0.86-0.90 (m, 11H), 0.99 (s, 3H), 1.00-1.18 (m, 5H), 1.19-1.20 (m, 3H), 1.20-1.52 (m, 11H), 1.57-1.62 (m, 1H), 1.69-1.82 (m, 4H), 1.93-2.00 (m, 2H), 2.16-2.18 (m, 1H), 2.33-2.39 (m, 2H), 3.43-3.51 (m, 1H), 3.71-3.76 (m, 1H), 4.18-4.27 (m, 2H), 4.43-4.47 (m, 1H), 5.30-5.31 (m, 1H).

(25*S*)-3β-(*tert*-Butyldimethylsilyloxy) cholest-5-en-26-oic Acid (7):

To a stirred solution of compound **6** (400 mg, 0.62 mmol) in THF: H_2O (3:1, 4 mL) were added 30% hydrogen peroxide (127 mg, 3.74 mmol) and lithium hydroxide monohydrate (52 mg, 1.24

mmol) at 0 ^oC and stirred for 3 h. The progress of the reaction was monitored by TLC; after completion of the reaction, the reaction mixture was quenched with aqueous sodium sulfite solution (15 mL), acidified with 1 N HCl solution (15 mL) and extracted with diethyl ether (2 x 20 mL). The combined organic extracts were washed with water (15 mL), dried over sodium sulphate, filtered and concentrated under reduced pressure to obtain the crude. The crude was purified through silica gel column chromatography using 10% EtOAc/ *n*-Hexane to afford compound **7** (300 mg, 91%) as white solid.

^1H NMR (400 MHz, $CDCl_3$): δ 0.05 (s, 6H), 0.66 (s, 3H), 0.88-0.91 (m, 14H), 0.99 (s, 4H), 1.09-1.13 (m, 5H), 1.18 (d, *J* = 7.2 Hz, 3H), 1.38-1.58 (m, 12H), 1.67-1.76 (m, 2H), 1.72-1.83 (m, 2H), 1.93-2.01 (m, 2H), 2.13-2.18 (m, 1H), 2.23-2.39 (m, 1H), 2.43-2.50 (m, 1H), 3.43-3.51 (m, 1H), 5.30-5.31 (m, 1H).

Methyl (25*S*)-3β-Hydroxycholesten-5-en-26-oate (8):

To a stirred solution of compound **7** (300 mg, 0.56 mmol) in methanol (4 mL) under inert atmosphere was added concentrated sulphuric acid (catalytic amount) at 0 ^oC; heated to reflux and stirred for 16 h. The progress of the reaction was monitored by TLC; after completion of the reaction, the volatiles were evaporated under reduced pressure. The residue was diluted with ice cold water (10 mL) and extracted with CH_2Cl_2 (2 x 10 mL). The combined organic extracts were washed with saturated $NaHCO_3$ solution (10 mL), dried over sodium sulfate, filtered and concentrated under reduced pressure to obtain the crude. The crude was purified through silica gel column chromatography using 20% EtOAc/ *n*-Hexane to afford compound **8** (180 mg, 74%) as white solid.

^1H NMR (400 MHz, $CDCl_3$): δ 0.67 (s, 3H), 0.90 (d, *J* = 6.8 Hz, 4H), 0.92-0.98 (m, 1H), 1.02-1.11 (m, 4H), 1.02-1.12 (m, 4H), 1.13-1.17 (m, 4H), 1.15-1.59 (m, 2H), 1.30-1.36 (m, 4H), 1.41-1.51 (m, 6H), 1.59-1.66 (m, 1H), 1.76-1.86 (m, 3H), 1.94-2.02 (m, 2H), 2.19-2.31 (m, 2H), 2.41-2.48 (m, 1H), 3.48-3.55 (m, 1H), 3.67 (s, 3H), 5.34-5.35 (m, 1H).

(25*S*)-Cholestenoic acid:

To a stirred solution of **8** (180 mg, 0.41 mmol) in THF: MeOH: H_2O (2:1:1, 4 mL) was added lithium hydroxide monohydrate (56 mg, 1.33 mmol) at RT and stirred for 16 h. The progress of the reaction was monitored by TLC; after completion of the reaction, the volatiles were evaporated under reduced pressure. The residue was diluted with water (10 mL), acidified with 1 N HCl solution (10 mL) and extracted with CH_2Cl_2 (2 x 10 mL). The combined organic layers were dried over sodium sulfate, filtered and concentrated under reduced pressure to obtain the

crude. The crude was triturated with acetonitrile (2 x 2 mL) to afford **(25S)-Cholestenoic acid** (30 mg, 52%) as white solid.

^{1}H NMR (400 MHz, $CDCl_3$): δ 0.67 (s, 3H), 0.91 (d, *J* = 6.4 Hz, 3H), 0.95-1.13 (m, 9H), 1.15 (d, *J* = 5.2 Hz, 3H), 1.21-1.31 (m, 3H), 1.34-1.73 (m, 13H), 1.78-1.87 (m, 3H), 1.93-2.03 (m, 2H), 2.18-2.34 (m, 2H), 2.44-2.52 (m, 1H), 3.48-3.56 (m, 1H), 5.34-5.35 (m, 1H).

Synthesis of Compound **6** (Table 1 in manuscript):

Scheme 3:

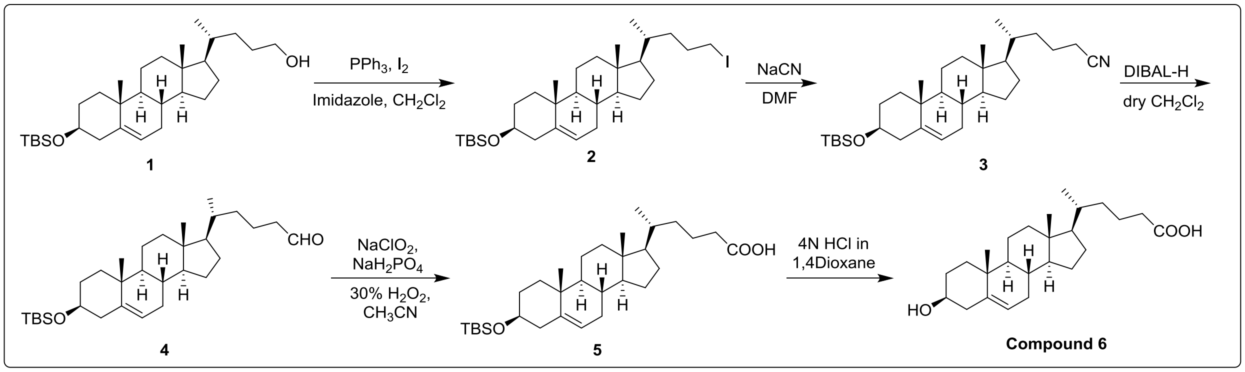

Experimental:

***tert*-butyl (((3*S*, 8*S*, 9*S*, 10*R*, 13*R*, 14*S*, 17*R*)-17-((*R*)-5-iodopentan-2-yl)-10, 13-dimethyl-2, 3, 4, 7, 8, 9, 10, 11, 12, 13, 14, 15, 16, 17-tetradecahydro-1*H*-cyclopenta [*a*] phenanthren-3-yl) oxy) dimethylsilane (2):**

To a stirred solution of triphenyl phosphine (2.8 g, 10.7 mmol) in dry CH_2Cl_2 (10 mL) under inert atmosphere were added iodine (2.2 g, 10.07 mmol) and imidazole (1.2 g, 17.9 mmol) at RT and stirred for 10 min. Then **1** (1.7 g, 3.58 mmol) in CH_2Cl_2 (10 mL) was added to the reaction mixture at RT and stirred for 2 h. The reaction was monitored by TLC; after completion of the reaction, the reaction mixture was diluted with saturated sodium thiosulphate solution (20 mL) and extracted with CH_2Cl_2 (2 x 25 mL). The combined organic extracts were washed with water (20 mL), dried over sodium sulphate, filtered and concentrated under reduced pressure to obtain the crude. The crude was purified through silica gel column chromatography using 2% EtOAc/*n*-Hexane to afford compound **2** (1.5 g, 72%) as white solid.

^{1}H NMR (500 MHz, $CDCl_3$): δ 0.05 (s, 6H), 0.67 (s, 3H), 0.86-1.12 (m, 13H), 1.26-1.29 (m, 4H), 1.40-1.54 (m, 6H), 1.69-1.72 (m, 4H), 1.76-1.88 (m, 3H), 1.95-2.00 (m, 6H), 2.15-2.17 (m, 2H), 2.25-2.27 (m, 2H), 3.11-3.19 (m, 2H), 3.46-3.48 (m, 1H), 5.30-5.31 (m, 1H).

(*R*)-5-((3*S*, 8*S*, 9*S*, 10*R*, 13*R*, 14*S*, 17*R*)-3-((*tert*-butyldimethylsilyl) oxy)-10, 13-dimethyl-2, 3, 4, 7, 8, 9, 10, 11, 12, 13, 14, 15, 16, 17-tetradecahydro-1*H*-cyclopenta [*a*] phenanthren-17-yl) hexanenitrile (3):

To a stirred solution of compound **2** (200 mg, 0.34 mmol) in DMF (2 mL) under inert atmosphere was added sodium cyanide (20 mg, 0.44 mmol) at RT; heated to 80 ^oC and stirred for 5 h. The reaction was monitored by TLC; after completion of the reaction, the reaction mixture was cooled to 0 ^oC, diluted with water (10 mL) and extracted with CH_2Cl_2 (2 x 10 mL). The combined

organic extracts were washed with water (10 mL), dried over sodium sulphate, filtered and concentrated under reduced pressure to obtain the crude. The crude was purified through silica gel column chromatography using 2% EtOAc/*n*-Hexane to afford compound **3** (140 mg, 85%) as white solid.

^{1}H NMR (500 MHz, $CDCl_3$): δ 0.05 (s, 6H), 0.68 (s, 3H), 0.89-1.32 (m, 13H), 1.38-1.52 (m, 7H), 1.70-1.75 (m, 6H), 1.81-1.86 (m, 4H), 1.95-2.00 (m, 4H), 2.15-2.17 (m, 6H), 2.24-2.34 (m, 2H), 3.45-3.50 (m, 1H), 5.30-5.32 (m, 1H).

(*R*)-5-((3*S*, 8*S* 9*S*, 10*R*, 13*R*, 14*S*, 17*R*)-3-((*tert*-butyldimethylsilyl) oxy)-10, 13-dimethyl-2, 3, 4, 7, 8, 9, 10, 11, 12, 13, 14, 15, 16, 17-tetradecahydro-1*H*-cyclopenta [*a*] phenanthren-17-yl) hexanal (4):

To a stirred solution of compound **3** (120 mg, 0.24 mmol) in dry CH_2Cl_2 (3 mL) under inert atmosphere was added DIBAL-H (88 mg, 6.21 mmol) at -78 ^oC and stirred for 3 h. The reaction was monitored by TLC; after completion of the reaction, the reaction mixture was diluted with saturated sodium potassium tartarate solution (10 mL) and extracted with CH_2Cl_2 (2 x 10 mL). The combined organic extracts were washed with water (10 mL), dried over sodium sulphate, filtered and concentrated under reduced pressure to obtain the crude compound **4** (90 mg) as white solid.

^{1}H NMR (400 MHz, $CDCl_3$): δ 0.05 (s, 6H), 0.67 (s, 3H), 0.81-0.91 (m, 10H), 0.94 (d, J = 6.8 Hz, 4H), 0.99 (s, 4H), 1.01-1.29 (m, 5H), 1.30-1.51 (m, 7H), 1.54-1.61 (m, 2H), 1.65-1.77 (m, 2H), 1.78-1.87 (m, 2H), 1.92-2.02 (m, 2H), 2.13-2.18 (m, 1H), 2.23-2.30 (m, 1H), 2.36-2.42 (m, 2H), 3.44-3.51 (m, 1H), 5.30-5.32 (m, 1H), 9.75-9.76 (m, 1H).

(*R*)-5-((3*S*, 8*S*, 9*S*, 10*R*, 13*R*, 14*S*, 17*R*)-3-((*tert*-butyldimethylsilyl) oxy)-10, 13-dimethyl-2, 3, 4, 7, 8, 9, 10, 11, 12, 13, 14, 15, 16, 17-tetradecahydro-1*H*-cyclopenta [*a*] phenanthren-17-yl) hexanoic acid (5):

To a stirred solution of compound **4** (90 mg, 0.18 mmol) in CH_3CN (0.1 mL) under inert atmosphere was added sodium dihydrogen phosphate (8.94 mg, 0.07 mmol), sodium chlorite (25 mg, 0.27 mmol), 30% hydrogen peroxide (31.6 mg, 0.93 mmol) at 0 ^oC; warmed to RT and stirred for 16 h. The reaction was monitored by TLC; after completion of reaction, the reaction mixture was diluted with water (15 mL) and extracted with EtOAc (2 x 20 mL). The combined organic extracts were washed with water (15 mL), dried over sodium sulphate, filtered and concentrated under reduced pressure to obtain the crude. The crude was purified through silica

gel column chromatography using 10% EtOAc/*n*-Hexane to afford compound **5** (30 mg, 32%) as white solid.

^{1}H NMR (400 MHz, $CDCl_3$): δ 0.06 (s, 6H), 0.67 (s, 3H), 0.87-0.89 (m, 10H), 0.93-0.94 (m, 4H), 0.99 (s, 4H), 1.06-1.14 (m, 4H), 1.25 (s, 5H), 1.39-1.54 (m, 6H), 1.67-1.77 (m, 4H), 1.94-2.07 (m, 2H), 2.13-2.18 (m, 1H), 2.23-2.29 (m, 3H), 3.45-3.50 (m, 1H), 5.30-5.31 (m, 1H).

(*R*)-5-((3*S*, 8*S*, 9*S*, 10*R*, 13*R*, 14*S*, 17*R*)-3-hydroxy-10, 13-dimethyl-2, 3, 4, 7, 8, 9, 10, 11, 12, 13, 14, 15, 16, 17-tetradecahydro-1*H*-cyclopenta [*a*] phenanthren-17-yl) hexanoic acid (6)

To the compound **5** (10 mg, 0.01 mmol) was added 4N HCl in 1, 4-Dioxane (1 mL) under inert atmosphere at 0 ^{o}C; warmed to RT and stirred for 3 h. The reaction was monitored by TLC; after completion of reaction, the volatiles were evaporated under reduced pressure. The residue was diluted with water (5 mL) and extracted with CH_2Cl_2 (2 x 10 mL). The combined organic extracts were washed saturated $NaHCO_3$ solution (2 x 5 mL) followed by water (5 mL), dried over sodium sulphate, filtered and concentrated under reduced pressure to obtain the crude. The crude was triturated with CH_3CN (2 x 3 mL) to afford **compound 6 (Table 1 in manuscript)** (3 mg, 39%) as a yellow solid.

^{1}H NMR (400 MHz, $CDCl_3$): δ 0.68 (s, 3H), 0.87-0.97 (m, 7H), 0.99-1.02 (m, 4H), 1.04-1.18 (m, 7H), 1.42-1.48 (m, 5H), 1.78-1.85 (m, 5H), 1.95-2.02 (m, 4H), 2.23-2.33 (m, 3H), 3.49-3.52 (m, 1H), 5.33-5.35 (m, 1H).

Synthesis of Compound **7** (Table 1 in Manuscript)

<u>Scheme 4:</u>

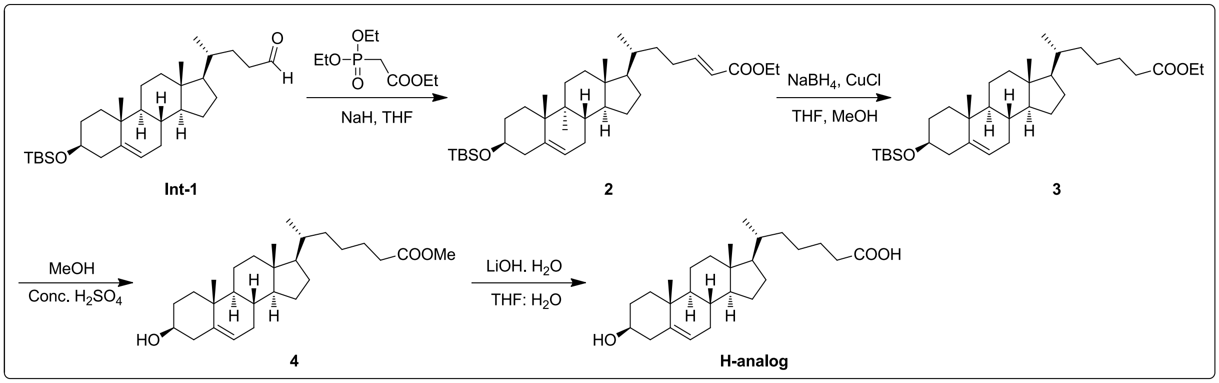

<u>Experimental:</u>

(*R*, *E*)-ethyl 6-((3*S*, 8*S*, 9*S*, 10*S*, 13*R*, 14*S*, 17*R*)-3-((*tert*-butyldimethylsilyl) oxy)-9, 10, 13-trimethyl-2, 3, 4, 7, 8, 9, 10, 11, 12, 13, 14, 15, 16, 17-tetradecahydro-1*H*-cyclopenta [*a*] phenanthren-17-yl) hept-2-enoate (2):

To a stirred solution of sodium hydride (51 mg, 1.27 mmol) in dry THF (1 mL) under inert atmosphere was added triethyl phosphonoacetate (213.5 mg, 0.95 mmol) at 0 ^{o}C and stirred for 1 h. Then **Int-1** (300 mg, 0.63 mmol) in THF (1 mL) was added to the reaction mixture at 0 ^{o}C and stirred for 1 h. The reaction was monitored by TLC; after completion of the reaction, the reaction mass was diluted with water (15 mL) and extracted with CH_2Cl_2 (2 x 20 mL). The combined organic extracts were washed with water (15 mL), dried over sodium sulphate, filtered and concentrated under reduced pressure to obtain the crude. The crude was purified through silica gel column chromatography using 5% EtOAc/ *n*-Hexane to afford compound **2** (200 mg, 58%) as white solid.

^{1}H NMR (500 MHz, $CDCl_3$): δ 0.06 (s, 6H), 0.68 (s, 3H), 0.96-0.98 (m, 1H), 0.99 (s, 3H), 1.00-1.02 (m, 3H), 1.03-1.06 (m, 6H), 1.07-1.10 (m, 3H), 1.13-1.23 (m, 8H), 1.26-1.30 (m, 6H), 1.41-1.61 (m, 6H), 1.68-1.73 (m, 1H), 1.79-1.84 (m, 2H), 1.95-2.01 (m, 2H), 2.09-2.18 (m, 2H), 2.24-2.29 (m, 2H), 3.46-3.50 (m, 1H), 4.18-4.20 (m, 2H), 5.31-5.33 (m, 1H), 5.79 (d, *J* = 15.5 Hz, 1H) , 6.94-6.99 (m, 1H).

(*R*)-ethyl 6-((3*S*, 8*S*, 9*S*, 10*R*, 13*R*, 14*S*, 17*R*)-3-((*tert*-butyldimethylsilyl) oxy)-10, 13-dimethyl-2, 3, 4, 7, 8, 9, 10, 11, 12, 13, 14, 15, 16, 17-tetradecahydro-1*H*-cyclopenta [*a*] phenanthren-17-yl) heptanoate (3):

To a stirred solution of compound **2** (100 mg, 0.18 mmol) in MeOH: THF (3: 7, 5 mL) under inert atmosphere were added copper chloride (13.5 mg, 0.13 mmol) and sodium borohydride (70 mg, 1.84 mmol) at 0 ^{o}C and stirred for 30 min; warmed to RT and stirred for 1 h. The reaction was monitored by TLC; after completion of the reaction, the reaction mass was filtered and the filtrate was diluted with water (15 mL) and extracted with CH_2Cl_2 (2 x 20 mL). The combined organic extracts were washed with water (15 mL), dried over sodium sulphate, filtered and concentrated under reduced pressure to obtain the crude. The crude was purified through silica gel column chromatography using 3% EtOAc/ *n*-Hexane to afford compound **3** (60 mg, 60%) as yellow solid.

^{1}H NMR (400 MHz, $CDCl_3$): δ 0.06 (s, 6H), 0.67 (s, 3H), 0.82-0.93 (m, 9H), 0.99 (s, 4H), 1.02-1.20 (m, 6H), 1.23-1.27 (m, 6H), 1.37-1.48 (m, 7H), 1.57-1.73 (m, 5H), 1.78-1.84 (m, 2H), 1.94-2.01 (m, 3H), 2.15-2.30 (m, 5H), 3.45-3.50 (m, 1H), 4.09-4.15 (m, 2H), 5.30-5.32 (m, 1H).

(*R*)-methyl 6-((3*S*, 8*S*, 9*S*, 10*R*, 13*R*, 14*S*, 17*R*)-3-hydroxy-10, 13-dimethyl-2, 3, 4, 7, 8, 9, 10, 11, 12, 13, 14, 15, 16, 17-tetradecahydro-1*H*-cyclopenta [*a*] phenanthren-17-yl) heptanoate (4):

To a stirred solution of compound **3** (60 mg, 0.09 mmol) in MeOH (2 mL) under inert atmosphere was added concentrated sulphuric acid (catalytic amount) at 0 ^{o}C; heated to reflux and stirred for 16 h. The reaction was monitored by TLC; after completion of the reaction, the volatiles were evaporated under reduced pressure. The residue was diluted with water (15 mL) and extracted with EtOAc (2 x 20 mL). The combined organic layers were washed with saturated $NaHCO_3$ solution (15 mL), washed with water (15 mL), dried over sodium sulphate, filtered and concentrated under reduced pressure to obtain the crude. The crude was purified through silica gel column chromatography using 15% EtOAc/ *n*-Hexane to afford compound **4** (30 mg, 65%) as white solid.

^{1}H NMR (400 MHz, $CDCl_3$): 0.67 (s, 3H), 0.90 (d, J = 6.8 Hz, 5H), 0.98-1.02 (m, 5H), 1.05-1.22 (m, 6H), 1.34-1.54 (m, 8H), 1.60-1.68 (m, 2H), 1.77-1.88 (m, 3H), 1.95-2.08 (m, 3H), 2.22-2.32 (m, 4H), 3.48-3.55 (m, 1H), 3.66 (s, 3H), 5.34-5.35 (m, 1H).

(*R*)-6-((3*S*, 8*S*, 9*S*, 10*R*, 13*R*, 14*S*, 17*R*)-3-hydroxy-10, 13-dimethyl-2, 3, 4, 7, 8, 9, 10, 11, 12, 13, 14, 15, 16, 17-tetradecahydro-1*H*-cyclopenta [*a*] phenanthren-17-yl) heptanoic acid (Compound 7):

To a stirred solution of compound **4** (30 mg, 0.07 mmol) in THF: H_2O (2: 1, 3 mL) was added lithium hydroxide monohydrate (10 mg, 0.21 mmol) at RT and stirred for 16 h. The reaction was

monitored by TLC; after completion of the reaction, the volatiles were evaporated under reduced pressure. The residue was diluted with water (10 mL) and extracted with CH_2Cl_2 (2 x 15 mL). The aqueous layer was acidified with 1 N HCl solution (10 mL) and extracted with CH_2Cl_2 (2 x 15 mL). The combined organic layers were washed with water (10 mL), dried over sodium sulphate, filtered and concentrated under reduced pressure to obtain the crude. The crude was triturated with CH_3CN (2 x 5 mL) to afford **compound 7** (12 mg, 41%) as a white solid.

^{1}H NMR (400 MHz, $CDCl_3$): δ 0.67 (s, 3H), 0.88-0.96 (m, 4H), 0.98-1.03 (m, 4H), 1.06-1.20 (m, 5H), 1.24-1.32 (m, 3H), 1.38-1.50 (m, 7H), 1.58-1.68 (m, 4H), 1.80-1.85 (m, 3H), 1.95-2.01 (m, 2H), 2.23-2.37 (m, 4H), 3.49-3.55 (m, 1H), 5.34-5.35 (m, 1H).

Synthesis of Compound **8** (Table 1 in Manuscript)

Scheme 5:

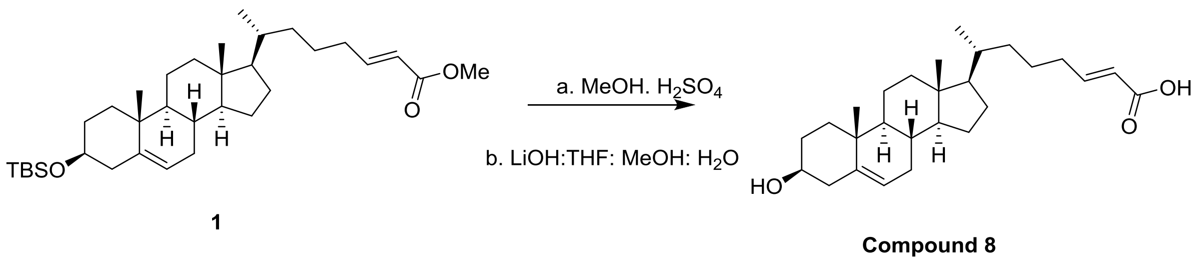

Experimental:

(*R*, *E*)-methyl 6-((3*S*, 8*S*, 9*S*, 10*R*, 13*R*, 14*S*, 17*R*)-3-hydroxy-10, 13-dimethyl-2, 3, 4, 7, 8, 9, 10, 11, 12, 13, 14, 15, 16, 17-tetradecahydro-1*H*-cyclopenta [*a*] phenanthren-17-yl) hept-2-enoate

To a stirred solution of **1** prepared by using analogous method described in Scheme 5 (50 mg, 0.09 mmol) in MeOH (1 mL) under inert atmosphere was added concentrated sulfuric acid (catalytic amount) at 0 ^{o}C; heated to reflux and stirred for 16 h. The reaction was monitored by TLC; after completion of the reaction, the volatiles were evaporated under reduced pressure. The residue was diluted with water (10 mL) and extracted with CH_2Cl_2 (2 x 15 mL). The combined organic extracts were washed with saturated $NaHCO_3$ solution (20 mL) followed by water (10 mL), dried over sodium sulphate, filtered and concentrated under reduced pressure to obtain the crude. The crude was purified through silica gel column chromatography using 8% EtOAc/ *n*-Hexane to afford compound **2** (30 mg, 47%) as white solid.

^{1}H NMR (400 MHz, $CDCl_3$): δ 0.68 (s, 3H), 0.72-0.91 (m, 2H), 0.92-0.98 (m, 3H), 0.99-1.04 (m, 2H), 1.05-1.23 (m, 5H), 1.24-1.34 (m, 5H), 1.37-1.52 (m, 4H), 1.58-1.64 (m, 2H), 1.78-1.90 (m, 2H), 1.91-2.06 (m, 3H), 2.08-2.14 (m, 1H), 2.18-2.32 (m, 3H), 3.51-3.53 (m, 1H), 3.72 (s, 3H), 5.34-5.35 (m, 1H), 5.79 (d, *J* = 18.4 Hz, 1H), 6.93-7.00 (m, 1H).

(*R*, *E*)-6-((3*S*, 8*S*, 9*S*, 10*R*, 13*R*, 14*S*, 17*R*)-3-((*tert*-butyldimethylsilyl) oxy)-10, 13-dimethyl-2, 3, 4, 7, 8, 9, 10, 11, 12, 13, 14, 15, 16, 17-tetradecahydro-1*H*-cyclopenta [*a*] phenanthren-17-yl) hept-2-enoic acid (compound 8):

To a stirred solution of compound **2** (30 mg, 0.07 mmol) in THF: MeOH: H_2O (2: 1: 1, 2 mL) was added lithium hydroxide monohydrate (9.1 mg, 0.21 mmol) at RT and stirred for 16 h. The

reaction was monitored by TLC; after completion of the reaction, the volatiles were evaporated under reduced pressure. The residue was diluted with water (10 mL) and extracted with CH_2Cl_2 (2 x 10 mL). The aqueous layer was acidified with 1 N HCl solution (10 mL) and extracted with CH_2Cl_2 (2 x 10 mL). The combined organic extracts were washed with water (15 mL), dried over sodium sulphate, filtered and concentrated under reduced pressure to obtain the crude. The crude was triturated with CH_3CN (2 x 5 mL) to afford **compound 8 (**12 mg, 42%) as white solid.

^{1}H NMR (400 MHz, $CDCl_3$): δ 0.67 (s, 3H), 0.68 (s, 4H), 0.78-0.87 (m, 1H), 0.88-0.95 (m, 1H), 1.02-1.04 (m, 1H), 1.05-1.12 (m, 4H), 1.13-1.21 (m, 5H), 1.25 (s, 3H), 1.41-1.63 (m, 4H), 1.78-1.87 (m, 3H), 1.95-2.04 (m, 2H), 2.08-2.18 (m, 1H), 2.23-2.32 (m, 3H), 3.48-3.50 (m, 1H), 5.34-5.35 (m, 1H), 5.82 (d, *J* = 15.6 Hz, 1H), 6.99-7.10 (m, 1H).

Synthesis of Difluoroanalog Compound **9** (Table 1 in Manuscript)

Scheme 6:

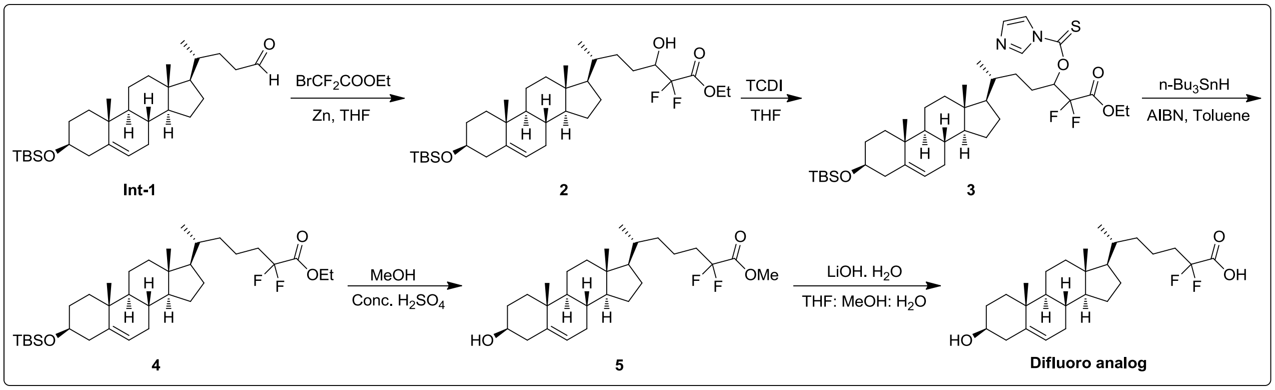

Experimental:

(6*R*)-ethyl 6-((3*S*, 8*S*, 9*S*, 10*R*, 13*R*, 14*S*, 17*R*)-3-((*tert*-butyldimethylsilyl) oxy)-10, 13-dimethyl-2, 3, 4, 7, 8, 9, 10, 11, 12, 13, 14, 15, 16, 17-tetradecahydro-1*H*-cyclopenta [*a*] phenanthren-17-yl)-2, 2-difluoro-3-hydroxyheptanoate (2):

A stirred solution of zinc dust (45.4 mg, 0.69 mmol) in THF (2 mL) under inert atmosphere was heated to reflux and were added **Int-1** (100 mg, 0.21 mmol) and ethyl 2-bromo-2,2-difluoroacetate (129 mg, 0.63 mmol) in THF (3 mL) and stirred for 45 min at reflux. The reaction was monitored by TLC; after completion of the reaction, the reaction mass was poured into 1 M solution of potassium hydrogen sulfate (10 mL) and extracted with EtOAc (2 x 20 mL). The combined organic extracts were washed with water (15 mL), dried over sodium sulphate, filtered and concentrated under reduced pressure to obtain the crude. The crude was purified through silica gel column chromatography using 5% EtOAc/ *n*-Hexane to afford compound **2** (80 mg, 56%) as an off-white solid.

^{1}H NMR (400 MHz, $CDCl_3$): δ 0.08 (s, 7H), 0.64-0.72 (m, 3H), 0.80-0.90 (m, 5H), 0.92-0.96 (m, 5H), 0.98-1.02 (m, 4H), 1.07-1.21 (m, 4H), 1.23-1.33 (m, 5H), 1.36 (t, J = 7.2 Hz, 5H), 1.43-1.53 (m, 3H), 1.56-1.64 (m, 3H), 1.69-1.84 (m, 4H), 1.93-2.03 (m, 3H), 2.14-2.31 (m, 2H), 3.45-3.50 (m, 1H), 3.93-3.98 (m, 1H), 4.36 (q, 2H), 5.30-5.32 (m, 1H).

(6*R*)-ethyl 3-((1*H*-imidazole-1-carbonothioyl) oxy)-6-((3*S*, 8*S*, 9*S*, 10*R*, 13*R*, 14*S*, 17*R*)-3-((*tert*-butyldimethylsilyl) oxy)-10, 13-dimethyl-2, 3, 4, 7, 8, 9, 10, 11, 12, 13, 14, 15, 16, 17-tetradecahydro-1*H*-cyclopenta [*a*] phenanthren-17-yl)-2, 2-difluoroheptanoate (3):

To a stirred solution of compound **2** (80 mg, 0.11 mmol) in THF (5 mL) under inert atmosphere was added TCDI (151.8 mg, 0.85 mmol) at RT; heated to reflux and stirred for 20 h. The

reaction was monitored by TLC; after completion of the reaction, the volatiles were removed under reduced pressure to obtain the crude. The crude was purified through silica gel column chromatography using 7% EtOAc/ *n*-Hexane to afford compound **3** (40 mg, 42%) as white solid.

^{1}H NMR (400 MHz, $CDCl_3$): δ 0.06 (s, 6H), 0.65 (d, *J* = 8.0 Hz, 3H), 0.80-0.90 (m, 14H), 0.92-0.96 (m, 4H), 0.99-1.02 (m, 4H), 1.08-1.20 (m, 7H), 1.40-1.52 (m, 5H), 1.70-1.82 (m, 4H), 1.90-2.02 (m, 3H), 2.16-2.32 (m, 2H), 3.44-3.50 (m, 1H), 4.27-4.37 (m, 2H), 5.30-5.31 (m, 1H), 6.05-6.10 (m, 1H), 7.05-7.06 (m, 1H), 7.60-7.61 (m, 1H), 8.32 (s, 1H).

(*R*)-ethyl 6-((3*S*, 8*S*, 9*S*, 10*R*, 13*R*, 14*S*, 17*R*)-3-((*tert*-butyldimethylsilyl) oxy)-10, 13-dimethyl-2, 3, 4, 7, 8, 9, 10, 11, 12, 13, 14, 15, 16, 17-tetradecahydro-1*H*-cyclopenta [*a*] phenanthren-17-yl)-2, 2-difluoroheptanoate (4):

To a stirred solution of compound **3** (40 mg, 0.05 mmol) in toluene (2 mL) under inert atmosphere were added tributyl tin hydride (33.16 mg, 0.11 mmol) and AIBN (0.94 mg, 0.005 mmol) at RT; heated to reflux and stirred for 30 min. The reaction was monitored by TLC; after completion of the reaction, the volatiles were removed under reduced pressure to obtain the crude. The crude was purified through silica gel column chromatography using 2% EtOAc/ *n*-Hexane to afford compound **4** (10 mg, 30%) as white solid.

^{1}H NMR (400 MHz, $CDCl_3$): δ 0.67 (s, 4H), 0.94-0.96 (m, 9H), 0.98-1.01 (m, 7H), 1.32-1.36 (m, 5H), 1.48-1.52 (m, 9H), 1.59-1.61 (m, 4H), 1.78-1.84 (m, 5H), 1.88-2.08 (m, 5H), 2.14-2.20 (m, 2H), 2.22-2.32 (m, 4H), 3.45-3.49 (m, 1H), 4.30-4.35 (m, 2H), 5.31-5.32 (m, 1H).

(*R*)-methyl 2, 2-difluoro-6-((3*S*, 8*S*, 9*S*, 10*R*, 13*R*, 14*S*, 17*R*)-3-hydroxy-10, 13-dimethyl-2, 3, 4, 7, 8, 9, 10, 11, 12, 13, 14, 15, 16, 17-tetradecahydro-1*H*-cyclopenta [*a*] phenanthren-17-yl) heptanoate (5):

To a stirred solution of compound **4** (50 mg, 0.08 mmol) in MeOH (2 mL) under inert atmosphere was added concentrated sulphuric acid (catalytic amount) at 0 ^{o}C; heated to reflux and stirred for 16 h. The reaction was monitored by TLC; after completion of the reaction, the volatiles were evaporated under reduced pressure. The residue was diluted with water (15 mL) and extracted with EtOAc (2 x 15 mL). The combined organic extracts were washed with saturated $NaHCO_3$ solution (15 mL), washed with water (15 mL), dried over sodium sulphate, filtered and concentrated under reduced pressure to obtain the crude. The crude was purified through silica gel column chromatography using 10% EtOAc/ *n*-Hexane to afford compound **5** (10 mg, 26%) as an off-white solid.

^{1}H NMR (400 MHz, $CDCl_3$): δ 0.06 (s, 4H), 0.67 (s, 3H), 0.92 (d, *J* = 6.4 Hz, 3H), 0.98-1.04 (m, 3H), 1.06-1.17 (m, 3H), 1.19-1.32 (m, 2H), 1.34-1.44 (m, 3H), 1.46-1.52 (m, 4H), 1.57-1.64 (m, 2H), 1.77-1.88 (m, 3H), 1.90-2.13 (m, 3H), 2.20-2.32 (m, 3H), 3.50-3.55 (m, 2H), 3.87 (s, 3H), 5.34-5.35 (m, 1H).

(*R*)-2, 2-difluoro-6-((3*S*, 8*S*, 9*S*, 10*R*, 13*R*, 14*S*, 17*R*)-3-hydroxy-10, 13-dimethyl-2, 3, 4, 7, 8, 9, 10, 11, 12, 13, 14, 15, 16, 17-tetradecahydro-1*H*-cyclopenta [*a*] phenanthren-17-yl) heptanoic acid (Difluoro analog):

To a stirred solution of compound **5** (10 mg, 0.02 mmol) in THF: MeOH: H_2O (2: 1: 1, 2 mL) was added lithium hydroxide monohydrate (2.78 mg, 0.06 mmol) at RT and stirred for 4 h. The reaction was monitored by TLC; after completion of the reaction, the volatiles were removed under reduced pressure. The residue was diluted with water (5 mL), acidified with 1 N HCl solution (5 mL) and extracted with EtOAc (2 x 10 mL). The combined organic extracts were washed with water (5 mL), dried over sodium sulphate, filtered and concentrated under reduced pressure to obtain the crude. The crude was triturated with CH_3CN (2 x 3 mL) to afford **difluoro analog compound 9** (4 mg, 42%) as an off-white solid.

^{1}H NMR (400 MHz, $CDCl_3$): δ 0.67 (s, 3H), 0.86-0.90 (m, 4H), 0.92-0.96 (m, 3H), 1.04 (s, 3H), 1.08-1.12 (m, 3H), 1.24-1.28 (m, 6H), 1.40-1.46 (m, 4H), 1.54-1.55 (m, 1H), 1.84-1.88 (m, 3H), 1.98-2.04 (m, 4H), 2.26-2.34 (m, 2H), 3.49-3.56 (m, 1H), 5.34-5.35 (m, 1H).

Synthesis Compound **10** (Table 1 in Manuscript)

Scheme 7:

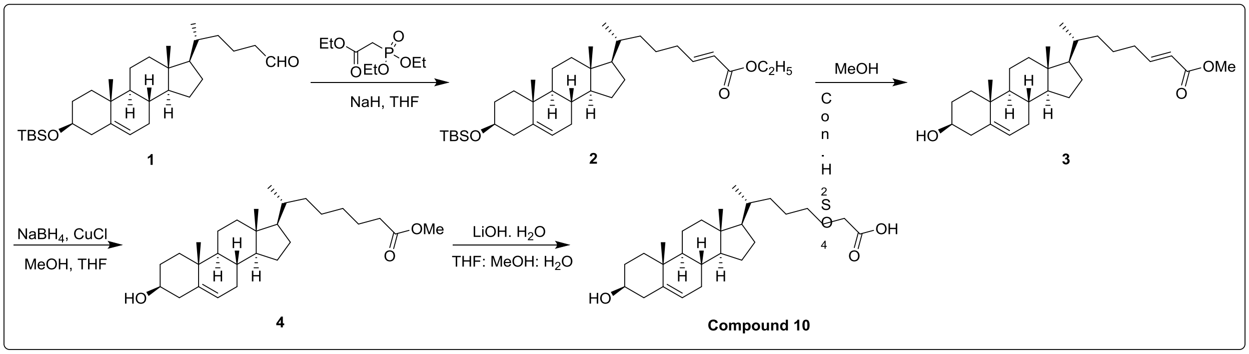

Experimental:

(*R*, *E*)-ethyl 7-((3*S*, 8*S*, 9*S*, 10*R*, 13*R*, 14*S*, 17*R*)-3-((*tert*-butyldimethylsilyl) oxy)-10, 13-dimethyl-2, 3, 4, 7, 8, 9, 10, 11, 12, 13, 14, 15, 16, 17-tetradecahydro-1*H*-cyclopenta [*a*] phenanthren-17-yl) oct-2-enoate (2):

To a stirred solution of sodiumhydide (56 mg, 1.39 mmol) in dry THF (3 mL) under inert atmosphere was added triethyl phosphonoacetate (235 mg, 1.04 mmol) at 0 ^{o}C and stirred for 1 h. Then **1** (340 mg, 0.69 mmol) in dry THF (3 mL) was added to the reaction mixture at 0 ^{o}C and stirred for 1 h. The reaction was monitored by TLC; after completion of the reaction, the reaction mixture was diluted with ice cold water (20 mL) and extracted with EtOAc (2 x 25 mL). The combined organic extracts were washed with water (20 mL), dried over sodium sulphate, filtered and concentrated under reduced pressure to obtain the crude. The crude compound was purified through silica gel column chromatography using 4% EtOAc/ *n*-Hexane to afford compound **2** (185 mg, 47%) as white solid.

^{1}H NMR (500 MHz, $CDCl_3$): δ 0.06 (s, 6H), 0.67 (s, 3H), 0.85-0.82 (m, 9H), 0.87 (s, 3H), 0.94-0.92 (m, 3H), 1.00-1.03 (m, 4H), 1.12-1.24 (m, 5H), 1.26-1.30 (m, 4H), 1.34-1.55 (m, 8H), 1.60-1.70 (m, 1H), 1.73-1.82 (m, 2H), 1.95-2.01 (m, 2H), 2.12-2.18 (m, 3H), 2.20-2.29 (m, 1H), 3.46-3.50 (m, 1H), 4.16-4.21 (m, 2H), 5.30-5.32 (m, 1H), 5.79-5.82 (m, 1H), 6.93-6.99 (m, 1H).

(*R*, *E*)-methyl 7-((3*S*, 8*S*, 9*S*, 10*R*, 13*R*, 14*S*, 17*R*)-3-hydroxy-10, 13-dimethyl-2, 3, 4, 7, 8, 9, 10, 11, 12, 13, 14, 15, 16, 17-tetradecahydro-1*H*-cyclopenta [*a*] phenanthren-17-yl) oct-2-enoate (3):

To a stirred solution of compound **2** (185 mg, 0.33 mmol) in methanol (3 mL) under inert atmosphere was added sulfuric acid (catalytic amount) at 0 ^{o}C; heated to reflux and stirred for 16 h. The reaction was monitored by TLC; after completion of the reaction, the reaction mixture was diluted with water (10 mL) and extracted with EtOAc (2 x 10 mL). The combined organic

extracts were washed with saturated $NaHCO_3$ solution (2 x20 mL) followed by water (10 mL), dried over sodium sulphate, filtered and concentrated under reduced pressure to obtain the crude. The crude was purified through silica gel column chromatography using 10% EtOAc/ *n*-Hexane to afford compound **3** (120 mg, 84%) as white solid.

^{1}H NMR (400 MHz, $CDCl_3$): δ 0.68 (s, 3H), 0.92 (d, *J* = 6.4 Hz, 6H), 1.01-1.21 (m, 8H), 1.26 (s, 3H), 1.37-1.52 (m, 8H), 1.77-1.87 (m, 3H), 1.96-2.06 (m, 2H), 2.10-2.32 (m, 4H), 3.49-3.55 (m, 1H), 3.71 (s, 3H), 5.35-5.36 (m, 1H), 5.80-5.84 (m, 1H), 6.94-7.01 (m, 1H).

(*R*)-methyl 7-((3*S*, 8*S*, 9*S*, 10*R*, 13*R*, 14*S*, 17*R*)-3-hydroxy-10, 13-dimethyl-2, 3, 4, 7, 8, 9, 10, 11, 12, 13, 14, 15, 16, 17-tetradecahydro-1*H*-cyclopenta [*a*] phenanthren-17-yl) octanoate (4):

To a stirred solution of compound **3** (185 mg, 0.33 mmol) in MeOH: THF (1:4, 5 mL) under inert atmosphere was added copper chloride (8.5 mg, 0.08 mmol) at RT. Then sodiumborohydride (44 mg, 1.16 mmol) was added to the reaction mass portion wise for 20 min at 0 ^oC; warmed to RT and stirred for 1 h. The reaction was monitored by TLC; after completion of the reaction, the reaction mixture was diluted with water (10 mL) and extracted with EtOAc (2 x 15 mL). The combined organic extracts were washed with water (10 mL), dried over sodium sulphate, filtered and concentrated under reduced pressure to obtain the crude compound **4** (40 mg) as white solid.

^{1}H NMR (400 MHz, $CDCl_3$): δ 0.67 (s, 3H), 0.80-0.97 (m, 7H), 0.98-1.02 (m, 4H), 1.07-1.22 (m, 7H), 1.28-1.51 (m, 8H), 1.57-1.63 (m, 3H), 1.79-1.86 (m, 3H), 1.94-2.02 (m, 2H), 2.20-2.32 (m, 4H), 3.48-3.52 (m, 1H), 3.52 (s, 3H), 5.34-5.35 (m, 1H).

(*R*)-7-((3*S*, 8*S*, 9*S*, 10*R*, 13*R*, 14*S*, 17*R*)-3-hydroxy-10, 13-dimethyl-2, 3, 4, 7, 8, 9, 10, 11, 12, 13, 14, 15, 16, 17-tetradecahydro-1*H*-cyclopenta [*a*] phenanthren-17-yl) octanoic acid (Compound 10):

To a stirred solution of compound **4** (40 mg, 0.09 mmol) in THF: MeOH: H_2O (2:1:1, 4 mL) under inert atmosphere was added lithium hydroxide monohydrate (11.7 mg, 0.27 mmol) at RT and stirred for 16 h. The reaction was monitored by TLC; after completion of the reaction, the reaction mixture was diluted with water (10 mL) and extracted with Et_2O (2 x 15 mL). Then the aqueous layer was acidified with 1 N HCl solution (10 mL) and extracted with CH_2Cl_2 (2 x 10 mL). The combined organic extracts were washed with water (10 mL), dried over sodium sulphate, filtered and concentrated under reduced pressure to obtain the crude. The crude was triturated with *n*-pentane (2 x 5 mL) to afford compound **10** (15 mg, 36%) as white solid.

^{1}H NMR (400 MHz, $CDCl_3$): δ 0.67 (s, 3H), 0.91 (d, *J* = 6.8 Hz, 3H), 1.00 (s, 4H), 1.06-1.11 (m, 4H), 1.17-1.21 (m, 2H), 1.21-1.31 (m, 4H), 1.22-1.29 (m, 4H), 1.31-1.42 (m, 3H), 1.44-1.53 (m, 4H), 1.60-1.65 (m, 2H), 1.77-1.86 (m, 3H), 1.94-2.03 (m, 2H), 2.23-2.29 (m, 2H), 2.32-2.37 (m, 2H), 3.53-3.51 (m, 1H), 5.34-5.35 (m, 1H).

Synthesis of Compound **11** (Table 1 in Manuscript)

Scheme 8:

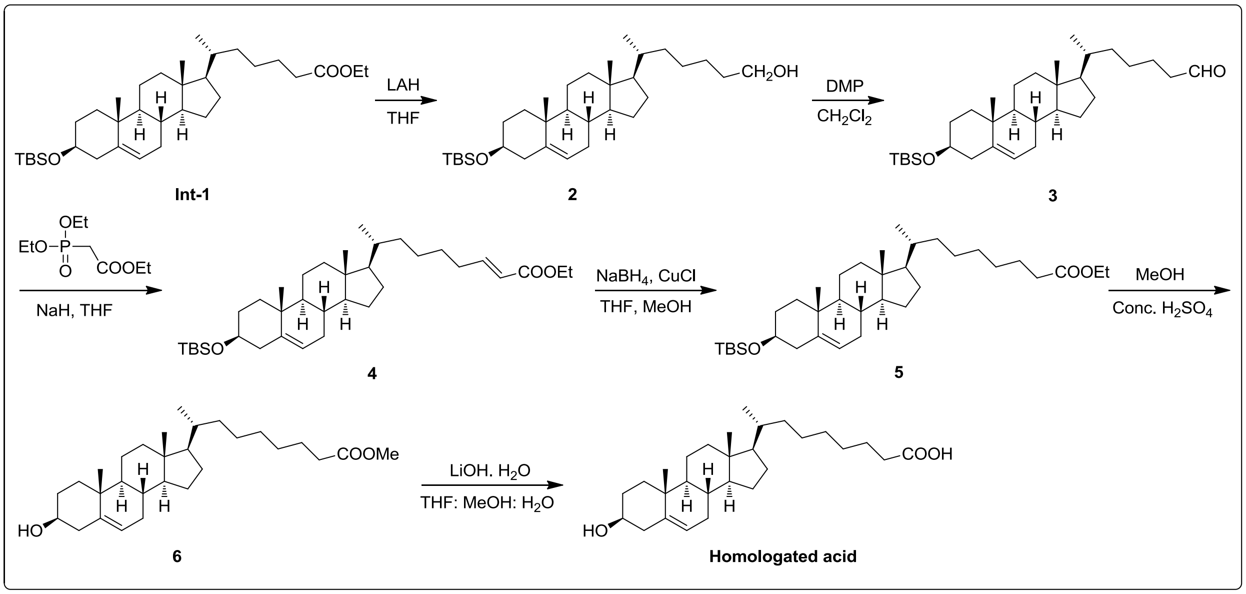

Experimental:

(*R*)-6-((3*S*, 8*S*, 9*S*, 10*R*, 13*R*, 14*S*, 17*R*)-3-((*tert*-butyldimethylsilyl) oxy)-10, 13-dimethyl-2, 3, 4, 7, 8, 9, 10, 11, 12, 13, 14, 15, 16, 17-tetradecahydro-1*H*-cyclopenta [*a*] phenanthren-17-yl) heptan-1-ol (2):

To a stirred solution of lithium aluminium hydride (52 mg, 1.37 mmol) in dry THF (20 mL) under inert atmosphere was added **Int-1** (500 mg, 0.91 mmol) at 0 ^{o}C; warmed to RT and stirred for 16 h. The reaction was monitored by TLC; after completion of the reaction, the reaction mass was quenched with ice cold water (20 mL) and extracted with EtOAc (2 x 20 mL). The combined organic extracts were washed with water (20 mL), dried over sodium sulphate, filtered and concentrated under reduced pressure to obtain the crude. The crude was purified through silica gel column chromatography using 5% EtOAc/ *n*-Hexane to afford compound **2** (400 mg, 86%) as white solid.

^{1}H NMR (400 MHz, $CDCl_3$): δ 0.05 (s, 6H), 0.67 (s, 3H), 0.84-0.96 (m, 13H), 0.99 (s, 5H), 1.06-1.12 (m, 4H), 1.14-1.28 (m, 6H), 1.32-1.48 (m, 8H), 1.58-1.60 (m, 2H), 1.69-1.84 (m, 3H), 1.94-2.01 (m, 2H), 2.14-2.26 (m, 2H), 3.45-3.50 (m, 1H), 3.61-3.66 (m, 2H), 5.30-5.32 (m, 1H).

(*R*)-6-((3*S*, 8*S*, 9*S*, 10*R*, 13*R*, 14*S*, 17*R*)-3-((*tert*-butyldimethylsilyl) oxy)-10, 13-dimethyl-2, 3, 4, 7, 8, 9, 10, 11, 12, 13, 14, 15, 16, 17-tetradecahydro-1*H*-cyclopenta [*a*] phenanthren-17-yl) heptanal (3):

To a stirred solution of compound **2** (100 mg, 0.19 mmol) in CH_2Cl_2 (10 mL) under inert atmosphere was added Dess-martin periodinane (253 mg, 0.59 mmol) at 0 ^oC, warmed to RT and stirred for 1 h. The reaction was monitored by TLC; after completion of the reaction, the reaction mass was filtered through celite and the filtrate was diluted with water (15 mL) and extracted with CH_2Cl_2 (2 x 15 mL). The combined organic extracts were washed with water (15 mL), dried over sodium sulphate, filtered and concentrated under reduced pressure to obtain the crude. The crude was purified through silica gel column chromatography using 2% EtOAc/ *n*-Hexane to afford compound **3** (50 mg, 50%) as white solid.

^{1}H NMR (400 MHz, $CDCl_3$): δ 0.06 (s, 6H), 0.67 (s, 3H), 0.80-0.88 (m, 9H), 0.91 (s, 5H), 0.98-1.03 (m, 4H), 1.04-1.14 (m, 5H), 1.16-1.30 (m, 5H), 1.32-1.44 (m, 5H), 1.58-1.64 (m, 2H), 1.68-1.74 (m, 1H), 1.78-1.86 (m, 2H), 1.92-2.04 (m, 2H), 2.11-2.21 (m, 1H), 2.22-2.30 (m, 1H), 2.39-2.43 (m, 2H), 3.43-3.49 (m, 1H), 5.30-5.32 (m, 1H), 9.75-9.76 (m, 1H).

(*R*, *E*)-ethyl 8-((3*S*, 8*S*, 9*S*, 10*R*, 13*R*, 14*S*, 17*R*)-3-((*tert*-butyldimethylsilyl) oxy)-10, 13-dimethyl-2, 3, 4, 7, 8, 9, 10, 11, 12, 13, 14, 15, 16, 17-tetradecahydro-1*H*-cyclopenta [*a*] phenanthren-17-yl) non-2-enoate (4):

To a stirred solution of sodium hydride (8 mg, 0.20 mmol) in dry THF (3 mL) under inert atmosphere was added triethyl phosphono acetate (34 mg, 0.15 mmol) at 0 ^oC and stirred for 1 h. Then compound **3** (50 mg, 0.10 mmol) in dry THF (2 mL) was added to the reaction mass at 0 ^oC and stirred for 1 h. The reaction was monitored by TLC; after completion of the reaction, the reaction mass was quenched with ice cold water (15 mL) and extracted with EtOAc (2 x 15 mL). The combined organic layers were washed with water (10 mL), dried over sodium sulphate, filtered and concentrated under reduced pressure to obtain the crude. The crude was purified through silica gel column chromatography using 2% EtOAc/ *n*-Hexane to afford compound **4** (50 mg, 87%) as white solid.

^{1}H NMR (400 MHz, $CDCl_3$): δ 0.05 (s, 6H), 0.67 (s, 3H), 0.80-0.94 (m, 9H), 0.98-1.02 (m, 4H), 1.04-1.24 (m, 7H), 1.28-1.32 (m, 4H), 1.34-1.54 (m, 10H), 1.57-1.63 (m, 4H), 1.68-1.74 (m, 1H), 1.78-1.86 (m, 2H), 1.90-2.04 (m, 2H), 2.10-2.34 (m, 4H), 3.44-3.51 (m, 1H), 4.18 (q, 2H), 5.30-5.32 (m, 1H), 5.78 (d, J = 18.4 Hz, 1H), 6.92-7.00 (m, 1H).

(*R*)-ethyl 8-((3*S*, 8*S*, 9*S*, 10*R*, 13*R*, 14*S*, 17*R*)-3-((*tert*-butyldimethylsilyl) oxy)-10, 13-dimethyl-2, 3, 4, 7, 8, 9, 10, 11, 12, 13, 14, 15, 16, 17-tetradecahydro-1*H*-cyclopenta [*a*] phenanthren-17-yl) nonanoate (5):

To a stirred solution of compound **4** (60 mg, 0.10 mmol) in MeOH: THF (7: 3, 5 mL) under inert atmosphere were added copper chloride (7.7 mg, 0.07 mmol) and sodium borohydride (40 mg, 1.05 mmol) at 0 ^{o}C; warmed to RT and stirred for 1 h. The reaction was monitored by TLC; after completion of the reaction, the reaction mass was quenched with ice cold water (15 mL), filtered and the filtrate was extracted with EtOAc (2 x 20 mL). The combined organic extracts were washed with water (15 mL), dried over sodium sulphate, filtered and concentrated under reduced pressure to obtain the crude. The crude was purified through silica gel column chromatography using 2% EtOAc/ *n*-Hexane to afford compound **5** (25 mg, 42%) as white solid.

^{1}H NMR (400 MHz, $CDCl_3$): δ 0.06 (s, 6H), 0.67 (s, 3H), 0.83-0.93 (m, 10H), 0.95-1.00 (m, 5H), 1.02-1.12 (m, 4H), 1.14-1.22 (m, 3H), 1.24-1.28 (m, 6H), 1.30-1.54 (m, 11H), 1.56-1.64 (m, 3H), 1.68-1.74 (m, 1H), 1.78-1.84 (m, 2H), 1.90-2.06 (m, 2H), 2.12-2.24 (m, 2H), 2.28 (t, *J* = 7.6 Hz, 2H), 3.43-3.51 (m, 1H), 4.12 (q, 2H), 5.30-5.32 (m, 1H).

(*R*)-methyl 8-((3*S*, 8*S*, 9*S*, 10*R*, 13*R*, 14*S*, 17*R*)-3-hydroxy-10, 13-dimethyl-2, 3, 4, 7, 8, 9, 10, 11, 12, 13, 14, 15, 16, 17-tetradecahydro-1*H*-cyclopenta [*a*] phenanthren-17-yl) nonanoate (6):

To a stirred solution of compound **5** (100 mg, 0.17 mmol) in MeOH (2 mL) under inert atmosphere was added concentrated sulphuric acid (catalytic amount) at 0 ^{o}C; heated to reflux and stirred for 2 h. The reaction was monitored by TLC; after completion of the reaction, the volatiles were evaporated under reduced pressure. The residue was diluted with water (15 mL) and extracted with EtOAc (2 x 20 mL). The combined organic layers were washed with saturated $NaHCO_3$ solution (15 mL), washed with water (15 mL), dried over sodium sulphate, filtered and concentrated under reduced pressure to obtain the crude. The crude was purified through silica gel column chromatography using 7% EtOAc/ *n*-Hexane to afford compound **6** (50 mg, 64%) as white solid.

^{1}H NMR (400 MHz, $CDCl_3$): δ 0.66 (s, 3H), 0.82-0.86 (m, 4H), 0.88-0.93 (m, 4H), 0.95-0.97 (m, 2H), 0.99-1.02 (m, 3H), 1.04-1.18 (m, 5H), 1.23-1.26 (m, 3H), 1.28 (s, 4H), 1.43-1.55 (m, 3H), 1.59-1.63 (m, 3H), 1.80-1.86 (m, 3H), 1.94-2.02 (m, 2H), 2.23-2.32 (m, 4H), 3.49-3.55 (m, 1H), 3.66 (s, 3H), 5.34-5.35 (m, 1H).

(*R*)-8-((3*S*, 8*S*, 9*S*, 10*R*, 13*R*, 14*S*, 17*R*)-3-hydroxy-10, 13-dimethyl-2, 3, 4, 7, 8, 9, 10, 11, 12, 13, 14, 15, 16, 17-tetradecahydro-1*H*-cyclopenta [*a*] phenanthren-17-yl) nonanoic acid (Compound 11):

To a stirred solution of compound **6** (50 mg, 0.11 mmol) in THF: MeOH: H_2O (2: 1: 1, 4 mL) was added lithium hydroxide monohydrate (13 mg, 0.33 mmol) at RT and stirred for 3 h. The reaction was monitored by TLC; after completion of the reaction, the reaction mass was diluted with water (15 mL), acidified with 1 N HCl solution (10 mL) and extracted with EtOAc (2 x 15 mL). The combined organic layers were washed with water (10 mL), dried over sodium sulphate, filtered and concentrated under reduced pressure to obtain the crude. The crude was triturated with CH_3CN (2 x 5 mL) to afford **compound 11** (20 mg, 42%) as white solid.

^{1}H NMR (400 MHz, $CDCl_3$): δ 0.66 (s, 3H), 0.97-0.99 (m, 4H), 1.02 (s, 4H), 1.03-1.29 (m, 5H), 1.32-1.47 (m, 8H), 1.49-1.67 (m, 12H), 1.80-1.86 (m, 3H), 2.23-2.28 (m, 2H), 2.35 (t, *J* = 7.6 Hz, 2H), 3.48-3.56 (m, 1H), 5.34-5.35 (m, 1H).

Synthesis of 3-Fluoro CA Analog Compound **12** (Table 2 in manuscript)

Scheme 9:

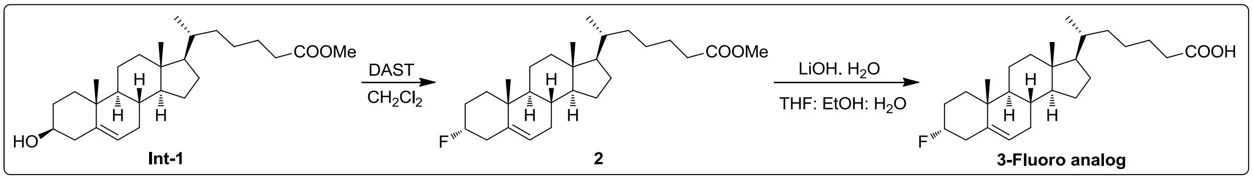

Experimental:

(*R*)-methyl 6-((3*R*, 8*S*, 9*S*, 10*R*, 13*R*, 14*S*, 17*R*)-3-fluoro-10, 13-dimethyl-2, 3, 4, 7, 8, 9, 10, 11, 12, 13, 14, 15, 16, 17-tetradecahydro-1*H*-cyclopenta [*a*] phenanthren-17-yl) heptanoate (2):

To a stirred solution of **Int-1** (25 mg, 0.06 mmol) in CH_2Cl_2 (3 mL) under inert atmosphere was added DAST (11.6 mg, 0.07 mmol) at -78 ^oC; warmed to RT and stirred for 15 min. The reaction was monitored by TLC; after completion of the reaction, the reaction mass was diluted with ice cold water (15 mL) and extracted with CH_2Cl_2 (2 x 15 mL). The combined organic extracts were washed with water (15 mL), dried over sodium sulphate, filtered and concentrated under reduced pressure to obtain the crude. The crude was purified through silica gel column chromatography using 2% EtOAc/ *n*-Hexane to afford compound **2** (10 mg, 40%) as white solid.
^{1}H NMR (400 MHz, $CDCl_3$): δ 0.67 (s, 3H), 0.90 (d, *J* = 6.4 Hz, 4H), 1.01-1.10 (m, 7H), 1.12-1.24 (m, 3H), 1.32-1.52 (m, 8H), 1.57-1.75 (m, 4H), 1.78-1.91 (m, 2H), 1.94-2.02 (m, 3H), 2.28-2.32 (m, 2H), 2.43 (t, *J* = 7.2 Hz, 2H), 3.66 (s, 3H), 4.22-4.42 (m, 1H), 5.38-5.39 (m, 1H).

(*R*)-6-((3*R*, 8*S*, 9*S*, 10*R*, 13*R*, 14*S*, 17*R*)-3-fluoro-10, 13-dimethyl-2, 3, 4, 7, 8, 9, 10, 11, 12, 13, 14, 15, 16, 17-tetradecahydro-1*H*-cyclopenta [*a*] phenanthren-17-yl) heptanoic acid (3-Fluoro analog):

To a stirred solution of compound **2** (10 mg, 0.02 mmol) in THF: EtOH: H_2O (2: 1: 1, 4 mL) under inert atmosphere was added lithium hydroxide monohydrate (3 mg, 0.07 mmol) at RT and stirred for 5 h. The reaction was monitored by TLC; after completion of the reaction, the volatiles were removed under reduced pressure. The residue was diluted with water (5 mL), acidified with 1 N HCl solution (5 mL) and extracted with EtOAc (2 x 10 mL). The combined organic extracts were washed with water (5 mL), dried over sodium sulphate, filtered and concentrated under reduced pressure to obtain the crude. The crude was triturated with *n*-pentane (2 x 3 mL) to afford **3-fluoro analog** (4 mg, 41%) as white solid.

^{1}H NMR (400 MHz, $CDCl_3$): δ 0.67 (s, 3H), 0.91 (d, *J* = 6.4 Hz, 4H), 1.00-1.04 (m, 4H), 1.06-1.20 (m, 4H), 1.24-1.30 (m, 3H), 1.34-1.44 (m, 4H), 1.46-1.50 (m, 3H), 1.60-1.72 (m, 4H), 1.78-1.88 (m, 2H), 1.90-2.06 (m, 3H), 2.35 (t, *J* = 7.2 Hz, 2H), 2.43 (t, *J* = 7.2 Hz, 2H), 4.30-4.46 (m, 1H), 5.38-5.39 (m, 1H).

Synthesis of 3-Deoxy Cholestenoic acid Compound **13** (Table 2 in manuscript)

Scheme 10:

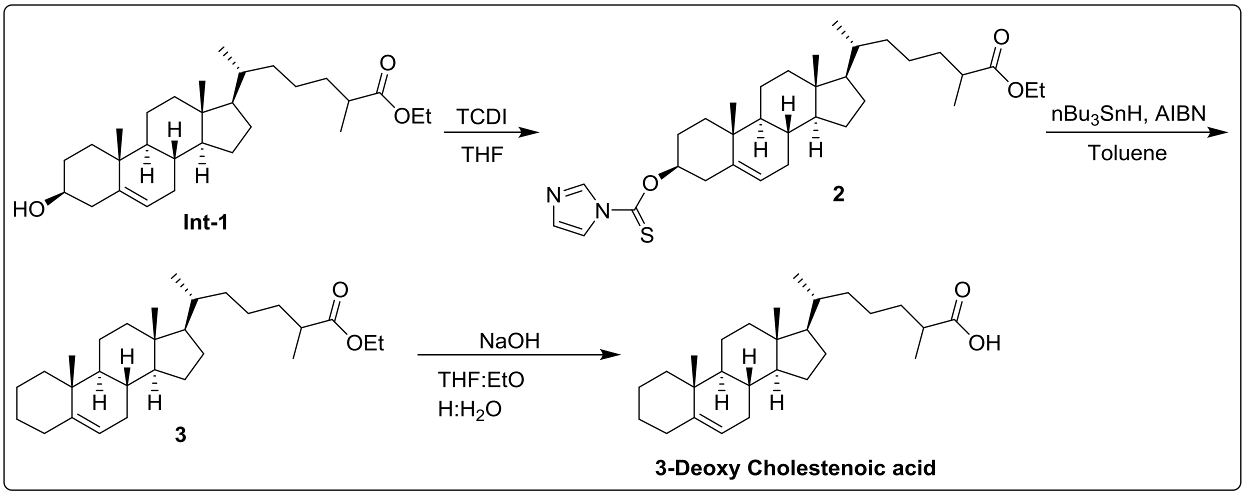

Experimental:

(6*R*)-ethyl 6-((3*S*, 8*S*, 9*S*, 10*R*, 13*R*, 14*S*, 17*R*)-3-((1*H*-imidazole-1-carbonothioyl) oxy)-10, 13-dimethyl-2, 3, 4, 7, 8, 9, 10, 11, 12, 13, 14, 15, 16, 17-tetradecahydro-1*H*-cyclopenta [*a*] phenanthren-17-yl)-2-methylheptanoate (2):

To a stirred solution of **Int-1** (800 mg, 1.80 mmol) in THF (10 mL) under inert atmosphere was added thiocarbonyldiimidazole (321 mg, 1.80 mmol) at RT; heated to reflux and stirred for 2 h. Then one more equivalent of TCDI (321 mg, 1.80 mmol) was added to the reaction mass and stirred at reflux for 16 h. The reaction was monitored by TLC; after completion of the reaction, the volatiles were removed under reduced pressure to obtain the crude. The crude was purified through silica gel column chromatography using 10-12% EtOAc/ *n*-Hexane to afford compound **2** (400 mg, 40%) as white solid.

^{1}H NMR (500 MHz, $CDCl_3$): δ 0.69 (d, *J* = 8.5 Hz, 3H), 0.89-0.91 (m, 3H), 0.99-1.02 (m, 3H), 1.10-1.12 (m, 5H), 1.16-1.22 (m, 6H), 1.26-1.32 (m, 6H), 1.36-1.42 (m, 4H), 1.48-1.62 (m, 6H), 1.78-1.86 (m, 2H), 1.98-2.04 (m, 3H), 2.22-2.26 (m, 1H), 2.39-2.43 (m, 1H), 4.10-4.13 (m, 2H), 5.32 (br s, 1H), 5.45-5.44 (br s, 1H), 7.10 (br s, 1H), 7.68 (br s, 1H), 8.49 (br s, 1H).

(6*R*)-ethyl 6-((8*S*, 9*S*, 10*R*, 13*R*, 14*S*, 17*R*)-10, 13-dimethyl-2, 3, 4, 7, 8, 9, 10, 11, 12, 13, 14, 15, 16, 17-tetradecahydro-1*H*-cyclopenta [*a*] phenanthren-17-yl)-2-methylheptanoate (3):

A stirred solution of tributyl tin hydride (525 mg, 1.80 mmol) in toluene (10 mL) under inert atmosphere was heated to reflux and then added compound **2** (250 mg, 0.45 mmol), AIBN (15 mg, 0.09 mmol) in toluene (10 mL) and stirred at reflux for 1 h; heated to 110 ^oC and stirred for 3 h. The reaction was monitored by TLC; after completion of the reaction, the volatiles were

removed under reduced pressure to obtain the crude. The crude was purified through silica gel column chromatography using 3% EtOAc/ *n*-Hexane to afford compound **3** (70 mg, 36%) as colorless syrup.

^{1}H NMR (500 MHz, $CDCl_3$): δ 0.67 (s, 3H), 0.87-0.90 (m, 2H), 0.93-0.95 (m, 2H), 0.97-0.99 (m, 3H), 1.06-1.11 (m, 5H), 1.13-1.15 (m, 3H), 1.19-1.20 (m, 1H), 1.26-1.28 (m, 3H), 1.30-1.40 (m, 10H), 1.46-1.52 (m, 3H), 1.60-1.61 (m, 1H), 1.72 (d, *J* = 11.5 Hz, 1H), 1.82 (d, *J* = 10.0 Hz, 2H), 1.93-2.00 (m, 3H), 2.22-2.26 (m, 1H), 2.38-2.41 (m, 1H), 4.10-4.14 (m, 2H), 5.26 (br s, 1H).

(6*R*)-6-((8*S*, 9*S*, 10*R*, 13*R*, 14*S*, 17*R*)-10, 13-dimethyl-2, 3, 4, 7, 8, 9, 10, 11, 12, 13, 14, 15, 16, 17-tetradecahydro-1*H*-cyclopenta [*a*] phenanthren-17-yl)-2-methylheptanoic acid (3-deoxycholestenoic acid):

To a stirred solution of compound **3** (70 mg, 0.16 mmol) in THF: EtOH: H_2O (3: 1: 1, 5 mL) was added sodium hydroxide (19.6 mg, 0.49 mmol) at RT; heated to reflux and stirred for 2 h. The reaction was monitored by TLC; after completion of the reaction, the volatiles were removed under reduced pressure. The residue was diluted with water (10 mL), acidified with 1 N HCl (5 mL) and extracted with EtOAc (2 x 20 mL). The combined organic extracts were washed with water (15 mL), dried over sodium sulphate, filtered and concentrated under reduced pressure to obtain the crude. The crude was purified through silica gel column chromatography using 10% EtOAc/ *n*-Hexane to afford 3-deoxycholestenoic acid (30 mg, 46%) as white solid.

^{1}H NMR (500 MHz, $CDCl_3$): δ 0.66 (d, *J* = 9.5 Hz, 3H), 0.89-0.92 (m, 3H), 0.97-1.00 (m, 4H), 1.01-1.16 (m, 5H), 1.18-1.21 (m, 5H), 1.36-1.48 (m, 7H), 1.50-1.62 (m, 6H), 1.65-1.84 (m, 4H), 1.94-2.01 (m, 3H), 2.21-2.24 (m, 1H), 2.46-2.49 (m, 1H), 5.27 (s, 1H).

Synthesis of 7-α-OH CA analog (Figure 2A and 5A)

Scheme 11:

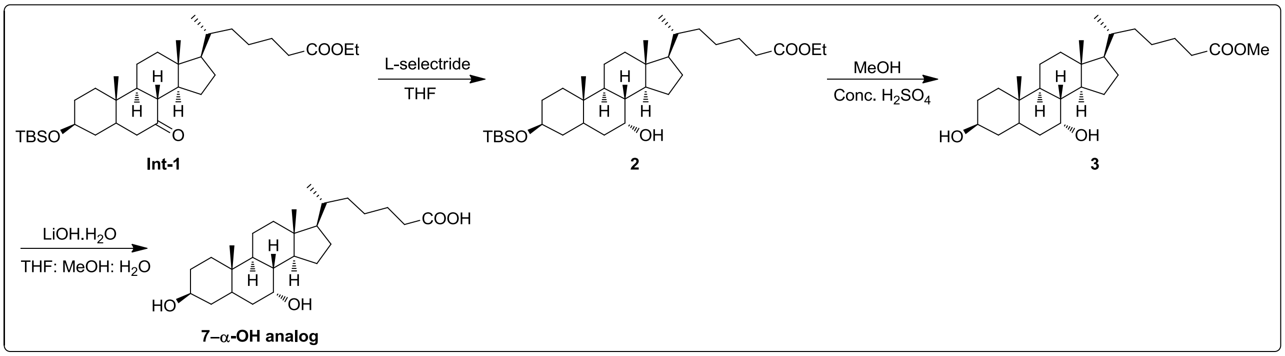

Experimental:

Synthesis of (6*R*)-ethyl 6-((3*S*, 7*R*, 8*R*, 9*S*, 10*S*, 13*R*, 14*S*, 17*R*)-3-((*tert*-butyldimethylsilyl) oxy)-7-hydroxy-10, 13-dimethylhexadecahydro-1*H*-cyclopenta [*a*] phenanthren-17-yl) heptanoate (2) (LNB No: SPM-MA1201-086):

To a stirred solution of **Int-1** (110 mg, 0.19 mmol) in THF (2 mL) under inert atmosphere was added 1 M L-selectride in THF (49 mg, 0.25 mmol) at -78 ^oC and stirred for 3 h. The reaction was monitored by TLC; after completion of the reaction, the reaction mass was diluted with 30% hydrogen peroxide solution (15 mL), saturated sodium bicarbonate solution (15 mL) at 0 ^oC; warmed to RT and stirred for 1 h. The reaction mixture was diluted with water (15 mL) and extracted with EtOAc (2 x 20 mL). The combined organic extracts were washed with water (15 mL), dried over sodium sulphate, filtered and concentrated under reduced pressure to obtain the crude. The crude was purified through silica gel column chromatography using 5% EtOAc/ *n*-Hexane to afford compound **2** (50 mg, 45%) as pale brown syrup.

^{1}H NMR (400 MHz, $CDCl_3$): δ 0.66 (s, 3H), 0.79-0.82 (m, 4H), 0.84-0.96 (m, 10H), 1.01-1.19 (m, 10H), 1.22-1.32 (m, 10H), 1.34-1.49 (m, 10H), 1.52-1.55 (m, 3H), 1.62-1.75 (m, 6H), 2.28-2.32 (m, 2H), 3.56-3.64 (m, 1H), 3.83-3.84 (m, 1H), 4.13 (q, 2H).

Synthesis of (6*R*)-methyl 6-((3*S*, 7*R*, 8*R*, 9*S*, 10*S*, 13*R*, 14*S*, 17*R*)-3, 7-dihydroxy-10, 13-dimethylhexadecahydro-1*H*-cyclopenta [*a*] phenanthren-17-yl) heptanoate (3) (LNB No: SPM-MA1202-047):

To a stirred solution of compound **2** (30 mg, 0.05 mmol) in MeOH (2 mL) under inert atmosphere was added concentrated sulphuric acid (catalytic amount) at RT; heated to reflux and stirred for 2 h. The reaction was monitored by TLC; after completion of the reaction, the volatiles were removed under reduced pressure. The residue was diluted with saturated sodium bicarbonate solution (10 mL) and extracted with EtOAc (2 x 15 mL). The combined organic extracts were washed with water (10 mL), dried over sodium sulphate, filtered and concentrated

under reduced pressure to obtain the crude. The crude was purified through silica gel column chromatography using 10% EtOAc/ *n*-Hexane to afford compound **3** (15 mg, 65%) as an off-white solid.

^{1}H NMR (400 MHz, $CDCl_3$): δ 0.66 (s, 3H), 0.80 (s, 3H), 0.92 (d, *J* = 6.4 Hz, 3H), 0.98-1.06 (m, 2H), 1.08-1.18 (m, 6H), 1.20-1.32 (m, 6H), 1.34-1.48 (m, 4H), 1.50-1.54 (m, 3H), 1.51-1.64 (m, 5H), 1.80-1.90 (m, 2H), 1.92-1.96 (m, 1H), 2.31 (t, *J* = 6.8 Hz, 2H), 3.26-3.30 (m, 1H), 3.60-3.63 (m, 1H), 3.64 (s, 3H), 3.82-3.84 (m, 1H).

Synthesis of UF-GSM1 (Target-1) & UF-GSM2 (Target-2)

<u>Scheme 12:</u>

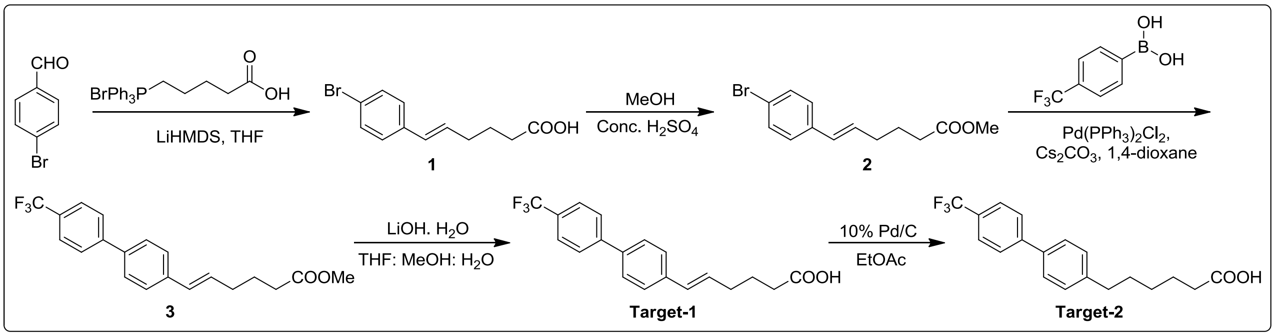

<u>Experimental:</u>

(*E*)-6-(4-bromophenyl) hex-5-enoic acid (1):

To a stirred solution of 5-(bromotriphenylphosphoranyl) pentanoic acid (1.2 g, 4.91 mmol) in dry THF (4 mL) under inert atmosphere was added LHMDS (1 M in THF) (6.48 mL, 6.84 mmol) at 0 ^{o}C; warmed to RT and stirred for 1 h. Then 4-bromobenzaldehyde (500 mg, 2.70 mmol) in dry THF (1 mL) was added to the reaction mass and stirred for 16 h. The progress of the reaction was monitored by TLC; after completion of the reaction, the reaction mixture was quenched with saturated ammonium chloride solution (25 mL) and extracted with CH_2Cl_2 (2 x 25 mL). The combined organic extracts were washed with water (20 mL), dried over sodium sulfate, filtered and concentrated under reduced pressure to obtain the crude. The crude was purified by acid-base treatment to afford compound **1** (300 mg, 41%) as colorless syrup.

^{1}H NMR (400 MHz, $CDCl_3$): δ 1.77-1.82 (m, 2H), 2.22-2.27 (m, 2H), 2.33-2.40 (m, 2H), 6.14-6.19 (m, 1H), 6.32 (d, *J* = 16.0 Hz, 1H), 7.18 (d, *J* = 8.4 Hz, 2H), 7.40-7.54 (m, 2H).

(*E*)-methyl 6-(4-bromophenyl) hex-5-enoate (2):

To a stirred solution of compound **1** (400 mg, 1.48 mmol) in MeOH (5 mL) under inert atmosphere was added concentrated sulphuric acid (catalytic amount) at 0 ^{o}C; warmed to RT and stirred for 16 h. The progress of the reaction was monitored by TLC; after completion of the reaction, the volatiles were removed under reduced pressure. The residue was diluted with water (20 mL) and extracted with EtOAc (2 x 25 mL). The combined organic extracts were washed with saturated $NaHCO_3$ solution (20 mL), dried over sodium sulphate, filtered and concentrated under reduced pressure to obtain the crude. The crude was purified through silica gel column chromatography using 10% EtOAc/ *n*-Hexane to afford compound **2** (260 mg, 55%) as yellow oil.

^{1}H NMR (400 MHz, $CDCl_3$): δ 1.79-1.83 (m, 2H), 2.23-2.25 (m, 2H), 2.32-2.37 (m, 2H), 3.66 (s, 3H), 6.14-6.20 (m, 1H), 6.33 (d, *J* = 15.6 Hz, 1H), 7.19 (d, *J* = 8.8 Hz, 2H), 7.38-7.45 (m, 2H).

(*E*)-methyl 6-(4'-(trifluoromethyl)-[1, 1'-biphenyl]-4-yl) hex-5-enoate (3):

To a stirred solution of compound **2** (100 mg, 0.35 mmol) in 1, 4-dioxane (5 mL) under inert atmosphere were added 4-trifluoro methyl benzene boronic acid (67 mg, 0.35 mmol), cesium carbonate (344 mg, 1.06 mmol) at RT and purged under argon for 15 min. Then Pd $(PPh_3)_2Cl_2$ (50 mg, 0.07 mmol) was added to the reaction mass at RT; heated to reflux and stirred for 16 h. The progress of the reaction was monitored by TLC; after completion of the reaction, the volatiles were removed under reduced pressure. The residue was diluted with water (15 mL) and extracted with EtOAc (2 x 20 mL). The combined organic extracts were washed with water (15 mL), dried over sodium sulphate, filtered and concentrated under reduced pressure to obtain the crude. The crude was purified through silica gel column chromatography using 8% EtOAc/ *n*-Hexane to afford compound **3** (48 mg, 39%) as white solid.

^{1}H NMR (400 MHz, $CDCl_3$): δ 1.84 (t, *J* = 7.2 Hz, 2H), 2.28-2.30 (m, 2H), 2.38 (t, *J* = 7.2 Hz, 2H), 3.67 (s, 3H), 6.26-6.28 (m, 1H), 6.44 (d, *J* = 15.6 Hz, 1H), 7.43 (d, *J* = 8.4 Hz, 2H), 7.45 (d, *J* = 8.0 Hz, 2H), 7.68 (s, 4H).

(*E*)-6-(4'-(trifluoromethyl)-[1, 1'-biphenyl]-4-yl) hex-5-enoic acid (Target-1):

To a stirred solution of compound **3** (48 mg, 0.13 mmol) in THF: MeOH: H_2O (2: 1: 1, 4 mL) was added lithium hydroxide monohydrate (17 mg, 0.41 mmol) at RT and stirred for 3 h. The progress of the reaction was monitored by TLC; after completion of the reaction, the volatiles were removed under reduced pressure. The residue was diluted with water (15 mL) and extracted with CH_2Cl_2 (2 x 15 mL). The aqueous layer was acidified with 1 N HCl solution (10 mL) and extracted with EtOAc (2 x 15 mL). The combined organic extracts were washed with water (15 mL), dried over sodium sulphate, filtered and concentrated under reduced pressure to obtain the crude. The crude was triturated with *n*-pentane (2 x 7 mL) to afford **Target-1** (25 mg, 54%) as an off-white solid.

^{1}H NMR (400 MHz, $CDCl_3$): δ 1.86-1.90 (m, 2H), 2.31-2.36 (m, 2H), 2.45 (t, *J* = 7.2 Hz, 2H), 6.23-6.31 (m, 1H), 6.47 (d, *J* = 15.6 Hz, 1H), 7.45 (d, *J* = 8.0 Hz, 2H), 7.56 (d, *J* = 8.4 Hz, 2H), 7.70 (s, 4H).

6-(4'-(trifluoromethyl)-[1, 1'-biphenyl]-4-yl) hexanoic acid (Target-2):

To a stirred solution of **Target-1** (20 mg, 0.05 mmol) in EtOAc (3 mL) under inert atmosphere was added 10% Pd/C (10 mg) at RT and stirred under hydrogen atmosphere (balloon pressure) for 4 h. The progress of the reaction was monitored by TLC; after completion of the reaction, the reaction mixture was filtered through celite and the filtrate was concentrated under reduced pressure to obtain the crude. The crude was triturated with *n*-pentane (2 x 5 mL) to afford **Target-2** (12 mg, 50%) as white solid.

^{1}H NMR (400 MHz, $CDCl_3$): δ 1.39-1.46 (m, 2H), 1.65-1.73 (m, 4H), 2.37 (t, *J* = 7.6 Hz, 2H), 2.67 (t, *J* = 7.6 Hz, 2H), 7.27 (d, *J* = 8.0 Hz, 2H), 7.51 (d, *J* = 8.0 Hz, 2H), 7.67 (s, 4H).

Synthesis of UF-GSM3 (Target-3) & UF-GSM4 (Target-4)

Scheme 13:

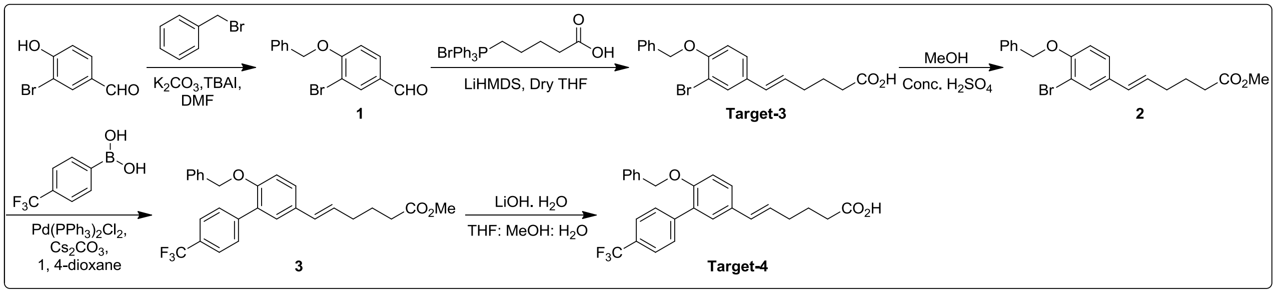

Experimental:

4-(benzyloxy)-3-bromobenzaldehyde (1):

To a stirred solution of 3-bromo-4-hydroxybenzaldehyde (1 g, 4.97 mmol) in DMF (10 mL) under inert atmosphere were added potassium carbonate (1.37 g, 9.95 mmol), TBAI (180 mg, 0.49 mmol) and benzyl bromide (0.72 mL, 9.94 mmol) at RT and stirred for 3 h. The progress of the reaction was monitored by TLC; after completion of the reaction, the reaction mixture was diluted with water (25 mL) to obtain the solid which was filtered, washed with water (20 mL), *n*-Hexane (20 mL) dried under reduced pressure to afford compound **1** (1.3 g, 90%) as colorless syrup.

^{1}H NMR (400 MHz, $CDCl_3$): δ 5.26 (s, 2H), 7.04 (d, *J* = 8.4 Hz, 1H), 7.35-7.47 (m, 5H), 7.78 (d, *J* = 8.4 Hz, 1H), 8.11 (s, 1H), 9.84 (s, 1H).

(*E*)-6-(4-(benzyloxy)-3-bromophenyl) hex-5-enoic acid (Taregt-3):

To a stirred solution of 5-(bromotriphenylphosphoranyl) pentanoic acid (2.37 g, 5.25 mmol) in dry THF (10 mL) under inert atmosphere was added LHMDS (1 M in THF) (10.5 mL, 10.50 mmol) at 0 ^oC; warmed to RT and stirred for 1 h. Then compound **1** (1.3 g, 4.46 mmol) in dry THF (5 mL) was added to the reaction mass and stirred for 3 h. The progress of the reaction was monitored by TLC; after completion of the reaction, the reaction mixture was quenched with aqueous 1 N HCl solution (25 mL) and extracted with EtOAc (2 x 25 mL). The combined organic extracts were washed with water (20 mL), dried over sodium sulfate, filtered and concentrated under reduced pressure to obtain the crude. The crude was purified by acid-base treatment to afford **Target-3** (1.2 g, 72%) as colorless syrup.

^{1}H NMR (500 MHz, $CDCl_3$): δ 1.79-1.84 (m, 2H), 2.23-2.37 (m, 2H), 2.34-2.41 (m, 2H), 5.14 (s, 2H), 6.01-6.07 (m, 1H), 6.26-6.29 (m, 1H), 6.85 (d, *J* = 8.5 Hz, 1H), 7.16 (d, *J* = 8.5 Hz, 1H), 7.25-7.32 (m, 1H), 7.36-7.39 (m, 1H), 7.46 (d, *J* = 7.5 Hz, 2H), 7.53-7.56 (m, 1H), 7.65-7.69 (m, 1H).

(*E*)-methyl 6-(4-(benzyloxy)-3-bromophenyl) hex-5-enoate (2):

To a stirred solution of **Target-3** (1.2 g, 3.44 mmol) in MeOH (15 mL) under inert atmosphere was added concentrated sulphuric acid (catalytic amount) at 0 ^{o}C; heated to reflux and stirred for 16 h. The progress of the reaction was monitored by TLC; after completion of the reaction, the volatiles were removed under reduced pressure. The residue was diluted with water (20 mL) and extracted with EtOAc (2 x 25 mL). The combined organic extracts were washed with saturated $NaHCO_3$ solution (20 mL), dried over sodium sulphate, filtered and concentrated under reduced pressure to obtain the crude. The crude was purified through silica gel column chromatography using 5% EtOAc/ *n*-Hexane to afford compound **2** (600 mg, 47%) as pale yellow liquid.

^{1}H NMR (500 MHz, $CDCl_3$): δ 1.77-1.83 (m, 2H), 2.21-2.25 (m, 2H), 2.30-2.37 (m, 2H), 3.67 (s, 3H), 5.16 (d, *J* = 7.0 Hz, 2H), 6.02-6.08 (m, 1H), 6.27 (d, *J* = 15.0 Hz, 1H), 6.86 (d, *J* = 8.5 Hz, 1H), 7.17 (d, *J* = 8.5 Hz, 1H), 7.30-7.33 (m, 1H), 7.37-7.40 (m, 2H), 7.47 (d, *J* = 7.0 Hz, 2H), 7.57 (s, 1H).

(*E*)-methyl 6-(6-(benzyloxy)-4'-(trifluoromethyl)-[1, 1'-biphenyl]-3-yl) hex-5-enoate (3):

To a stirred solution of compound **2** (600 mg, 1.54 mmol) in 1, 4-dioxane (10 mL) under inert atmosphere were added 4-trifluoro methyl benzene boronic acid (323 mg, 1.70 mmol), cesium carbonate (1.5 g, 4.63 mmol) at RT and purged under argon for 15 min. Then $Pd(PPh_3)_2Cl_2$ (217 mg, 0.30 mmol) was added to the reaction mass at RT; heated to reflux and stirred for 16 h. The progress of the reaction was monitored by TLC; after completion of the reaction, the volatiles were removed under reduced pressure. The residue was diluted with EtOAc (15 mL) and filtered through celite and the filtrate was concentrated under reduced pressure to obtain the crude. The crude was purified through silica gel column chromatography using 8% EtOAc/ *n*-Hexane to afford compound **3** (500 mg, 71%) as yellow liquid.

^{1}H NMR (400 MHz, $CDCl_3$): δ 1.78-1.85 (m, 2H), 2.22-2.28 (m, 2H), 2.35-2.39 (m, 2H), 3.67 (s, 3H), 5.09 (s, 2H), 6.07-6.13 (m, 1H), 6.38 (d, *J* = 16.0 Hz, 1H), 6.98 (d, *J* = 8.4 Hz, 1H), 7.27-7.35 (m, 7H), 7.63-7.69 (m, 4H).

(*E*)-6-(6-(benzyloxy)-4'-(trifluoromethyl)-[1, 1'-biphenyl]-3-yl) hex-5-enoic acid (Target-4):

To a stirred solution of compound **3** (50 mg, 0.11 mmol) in THF: MeOH: H_2O (2: 1: 1, 4 mL) was added lithium hydroxide monohydrate (14 mg, 0.33 mmol) at RT and stirred for 3 h. The progress of the reaction was monitored by TLC; after completion of the reaction, the volatiles were removed under reduced pressure. The residue was diluted with water (15 mL) and

extracted with CH_2Cl_2 (2 x 15 mL). The aqueous layer was acidified with 1 N HCl solution (10 mL) and extracted with EtOAc (2 x 15 mL). The combined organic extracts were washed with water (15 mL), dried over sodium sulphate, filtered and concentrated under reduced pressure to obtain the crude. The crude was triturated with *n*-pentane (2 x 7 mL) to afford **Target-4** (25 mg, 52%) as white solid.

^{1}H NMR (500 MHz, $CDCl_3$): δ 1.81-1.84 (m, 2H), 2.25-2.29 (m, 2H), 2.39-2.42 (m, 2H), 5.08 (s, 2H), 6.07-6.10 (m, 1H), 6.39 (d, *J* = 15.5 Hz, 1H), 6.98 (d, *J* = 8.5 Hz, 1H), 7.27-7.32 (m, 7H), 7.63-7.68 (m, 4H).

Synthesis of UF-GSM5 (Target-6)

Scheme 14:

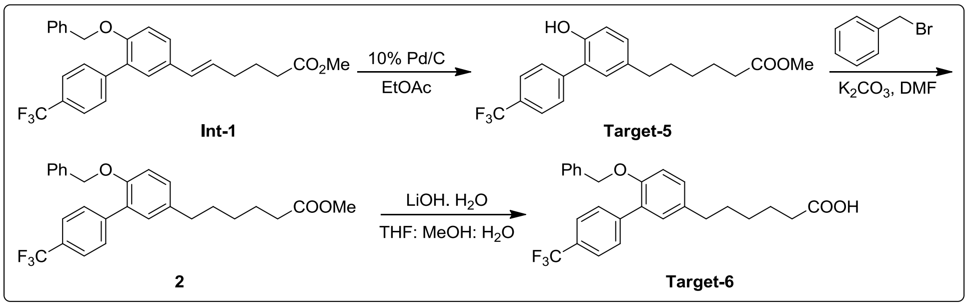

Experimental:

methyl 6-(6-hydroxy-4'-(trifluoromethyl)-[1, 1'-biphenyl]-3-yl) hexanoate (Target-5):

To a stirred solution of **Int-1** (450 mg, 0.99 mmol) in EtOAc (10 mL) under inert atmosphere was added 10% Pd/C (200 mg) at RT and stirred under hydrogen atmosphere (balloon pressure) for 16 h. The progress of the reaction was monitored by TLC; after completion of the reaction, the reaction mixture was filtered through celite and the filtrate was concentrated under reduced pressure to obtain the crude. The crude was triturated with *n*-pentane (2 x 10 mL) to afford **Target-5** (260 mg, 72%) as an off-white solid.

^{1}H NMR (400 MHz, $CDCl_3$): δ 1.35-1.40 (m, 2H), 1.61-1.68 (m, 4H), 2.31 (t, *J* = 7.2 Hz, 2H), 2.58 (t, *J* = 7.6 Hz, 2H), 3.65 (s, 3H), 4.85 (s, 1H), 6.87 (d, *J* = 8.0 Hz, 1H), 7.05-7.09 (m, 2H), 7.62 (d, *J* = 8.0 Hz, 2H), 7.72 (d, *J* = 8.0 Hz, 2H).

methyl 6-(6-(benzyloxy)-4'-(trifluoromethyl)-[1, 1'-biphenyl]-3-yl) hexanoate (2):

To a stirred solution of **Target-5** (100 mg, 0.27 mmol) in DMF (10 mL) under inert atmosphere were added potassium carbonate (75 mg, 0.54 mmol), TBAI (10 mg, 0.02 mmol) and benzyl bromide (0.4 mL, 0.32 mmol) at RT and stirred for 5 h. The progress of the reaction was monitored by TLC; after completion of the reaction, the reaction mixture was diluted with water (25 mL) and extracted with ether (2 x 25 mL). The combined organic extracts were washed with water (20 mL), dried over sodium sulfate, filtered and concentrated under reduced pressure to obtain the crude. The crude was purified through silica gel column chromatography using 7% EtOAc/ *n*-Hexane to afford compound **2** (100 mg, 80%) as pale brown syrup.

^{1}H NMR (400 MHz, $CDCl_3$): δ 1.35-1.41 (m, 2H), 1.60-1.70 (m, 4H), 2.30 (t, *J* = 7.6 Hz, 2H), 2.58-2.62 (m, 2H), 3.65 (s, 3H), 5.05 (s, 2H), 6.96 (d, *J* = 8.0 Hz, 1H), 7.10-7.13 (m, 2H), 7.27-7.32 (m, 5H), 7.62-7.68 (m, 4H).

6-(6-(benzyloxy)-4'-(trifluoromethyl)-[1, 1'-biphenyl]-3-yl) hexanoic acid (Target-6) :

To a stirred solution of compound **2** (100 mg, 0.21 mmol) in THF: MeOH: H_2O (2: 1: 1, 4 mL) was added lithium hydroxide monohydrate (27 mg, 0.65 mmol) at RT and stirred for 3 h. The progress of the reaction was monitored by TLC; after completion of the reaction, the volatiles were removed under reduced pressure. The residue was diluted with water (10 mL) and extracted with ether (2 x 10 mL). The aqueous layer was acidified with 1 N HCl solution (10 mL) and extracted with EtOAc (2 x 15 mL). The combined organic extracts were washed with water (10 mL), dried over sodium sulphate, filtered and concentrated under reduced pressure to obtain the crude. The crude was triturated with *n*-pentane (2 x 5 mL) to afford **Target-6** (40 mg, 41%) as pale yellow solid.

^{1}H NMR (500 MHz, $CDCl_3$): δ 1.40-1.43 (m, 2H), 1.62-1.70 (m, 4H), 2.36 (t, *J* = 7.5 Hz, 2H), 2.61 (t, *J* = 7.0 Hz, 2H), 5.06 (s, 2H), 6.97 (d, *J* = 8.0 Hz, 1H), 7.13 (d, *J* = 8.8 Hz, 2H), 7.30-7.33 (m, 5H), 7.64 (d, *J* = 8.5 Hz, 2H), 7.68 (d, *J* = 8.5 Hz, 2H).
